# Association of the Long Non-coding RNA Steroid Receptor RNA Activator (SRA) with TrxG and PRC2 Complexes

**DOI:** 10.1371/journal.pgen.1005615

**Published:** 2015-10-23

**Authors:** Patompon Wongtrakoongate, Gregory Riddick, Suthat Fucharoen, Gary Felsenfeld

**Affiliations:** 1 Laboratory of Molecular Biology, National Institute of Diabetes and Digestive and Kidney Diseases, National Institutes of Health, Bethesda, Maryland, United States of America; 2 Department of Biochemistry, Faculty of Science, Mahidol University, Bangkok, Thailand; 3 Thalassemia Research Center, Institute of Molecular Biosciences, Mahidol University, Nakhonpathom, Thailand; Massachusetts General Hospital, Howard Hughes Medical Institute, UNITED STATES

## Abstract

Long non-coding RNAs (lncRNAs) have been recognized as key players in transcriptional regulation. We show that the lncRNA steroid receptor RNA activator (SRA) participates in regulation through complex formation with trithorax group (TrxG) and polycomb repressive complex 2 (PRC2) complexes. Binding of the SRA-associated RNA helicase p68 preferentially stabilizes complex formation between SRA and a TrxG complex but not PRC2. In human pluripotent stem cells NTERA2, SRA binding sites that are also occupied by p68 are significantly enriched for H3K4 trimethylation. Consistent with its ability to interact with TrxG and PRC2 complexes, some SRA binding sites in human pluripotent stem cells overlap with bivalent domains. CTCF sites associated with SRA appear also to be enriched for bivalent modifications. We identify NANOG as a transcription factor directly interacting with SRA and co-localizing with it genome-wide in NTERA2. Further, we show that SRA is important for maintaining the stem cell state and for reprogramming of human fibroblasts to achieve the pluripotent state. Our results suggest a mechanism whereby the lncRNA SRA interacts with either TrxG or PRC2. These complexes may then be recruited by various DNA binding factors to deliver either activating or silencing signals, or both, to establish bivalent domains.

## Author Summary

Long non-coding RNAs (lncRNAs) can play an important role in regulation of gene expression. In a number of cases, individual lncRNAs have been shown to interact with either the trithorax group (TrxG) or polycomb repressive complex 2 (PRC2) protein complexes, which deliver histone modifications associated respectively with transcriptionally active or inactive chromatin. Here we show that the lncRNA, SRA, unusually forms complexes with both TrxG and PRC2 complexes. Consistent with this property, some SRA binding sites in human pluripotent stem cells overlap with bivalent domains, which carry both kinds of histone modifications. We find that SRA complexed with the helicase protein, p68, shows enhanced binding of TrxG complex, but not of PRC2. This is reflected in genome wide enriched ‘activating’ histone modifications at SRA sites also occupied by p68. We show that in human pluripotent stem cells SRA also interacts with NANOG, a principal determinant of pluripotency, and is important for maintenance of the pluripotent state. SRA may be involved in the delivery of histone modifications associated with either activation or silencing of gene expression, and in some cases could deliver both.

## Introduction

Histone H3 modifications involving lysine 4 trimethylation (H3K4me3) and lysine 27 trimethylation (H3K27me3) represent activating and repressive histone marks, respectively. However, when present together, as they are in bivalent sites, they mark genes that are poised for induction. Genes carrying the bivalent modification include those involved in differentiation of pluripotent stem cells. Two distinct histone modification machineries, associated with the trithorax group (TrxG) complex and with polycomb repressive complex 2 (PRC2), are responsible for methylating H3K4 and H3K27, respectively. TrxG complexes comprise at least four protein components, WDR5, RBBP5, ASH2L and an H3K4 methyltransferase such as MLL (MLL1-4), whereas EZH2, EED and SUZ12 are core components of PRC2. Establishment of bivalent domains involves delivery of these two complexes to their target regions. Both MLL1 and MLL2 containing complexes deliver trimethyl marks to H3K4, and MLL2 is required for this modification at bivalent sites in mouse embryonic stem cells [1, 2]. CpG islands (CGIs) have been reported to play an important role in recruitment of TrxG and PRC2 complexes via several CGI-binding proteins [3]. In addition, TrxG complex has been shown to be recruited directly by DNA sequence-specific transcription factors Oct4 [4] and estrogen receptor α (ERα) [5]. Similarly, at least one component of the PRC2 complex, SUZ12, can be targeted directly by the transcription factor CTCF [6]. Moreover, PRC2 target genes can recruit the complex through interaction with short RNAs transcribed from the 5’ ends of those genes [7–9]. We note that although under some solvent conditions PRC2 may exhibit non-specific interaction with RNA [9, 10], the experiments reported here, carried out in nuclear extracts or in PBS buffer, clearly show specificity for SRA.

A growing number of long non-coding RNAs (lncRNAs) have been implicated in recruitment of TrxG or PRC2 complexes to their target genes [11]. Two groups of lncRNAs may be categorized according to whether TrxG or PRC2 complexes bind to them, defining activating and repressive lncRNAs respectively. The first category of activating lncRNAs, which recruit TrxG complexes to their target genes via WDR5, includes Hottip [12], NeST [13] and NANCI [14]. In contrast, examples of lncRNAs belonging to the second category of repressive lncRNAs, which recruit PRC2 complex to its binding sites, are Xist [15], Hotair [16] and Braveheart [17]. The complete PRC2 complex has been shown to bind highly selectively to Hotair and RepA/Xist, as compared with control RNA [18]. Of the three core components of PRC2 comprising EZH2, SUZ12 and EED, it has been shown recently that EZH2 and SUZ12 possess a high affinity for RNA binding, whereas EED helps to increase RNA binding specificity to the complex [18].

Recently, a novel technique, Chromatin Isolation by RNA Purification (ChIRP), has provided a powerful method to map the location of lncRNAs genome-wide [19]. Using this technique, the lncRNA HOTAIR was shown to co-localize with the PRC2 complex and H3K27me3 genome-wide, supporting its functional role in tethering PRC2 to target genes. Similar techniques have been utilized to map the distribution of the lncRNA Xist, which also has a domain that recruits the PRC2 complex, along the X chromosome [20, 21]. Although these and other lncRNA species have been shown to deliver either “activating” or “silencing” histone modifications, it is not clear whether they can function coordinately to create bivalent domains.

The lncRNA steroid receptor RNA activator (SRA) can be recruited to DNA through interactions with proteins that bind either directly or indirectly to DNA [22]. For example, SRA has been shown to interact directly with ERα [23], which binds to specific DNA sequences, and to co-activate ERα target genes [24]. It also forms a complex with the DEAD box RNA helicase p68, which in turn interacts with the DNA binding protein MyoD [25]. We have reported previously that SRA and p68 form a complex with CTCF and are crucial for insulator function of CTCF at the *IGF2-H19* locus [26]. Furthermore, it has been shown that SRA can interact with EZH2 [27], suggesting that it might be involved in silencing functions associated with the PRC2 complex. In addition, SRA also interacts with HP1 gamma and LSD1 to repress progesterone receptor target genes [28]. In possible contradiction of that repressive function is the observation that knockdown of *SRA* in HeLa cells results in decreased expression of the majority of significantly changed genes [29].

In this study, we show that the lncRNA SRA is capable of binding TrxG and PRC2. Direct interaction with the complexes is specific for sense SRA as compared with the control, its anti-sense counterpart. SRA-p68 interaction strengthens recruitment of a TrxG complex but does not affect PRC2. We find that CTCF binding sites that are also occupied by SRA, are more likely to have bivalent marks. We also find that SRA/p68 associates with NANOG, a master transcription factor in pluripotent stem cells. These results show that SRA can associate with TrxG and PRC2 complexes to deliver either activating or repressive histone modifications, and that the choice can be modulated by proteins with which it associates. They also suggest a mechanism in which the bivalent state may be controlled at certain sites, including those occupied by NANOG, through recruitment of SRA and its associated histone modifying enzymes in pluripotent stem cells.

## Results

### SRA interacts with TrxG and PRC2 complexes

**Fig 1:**
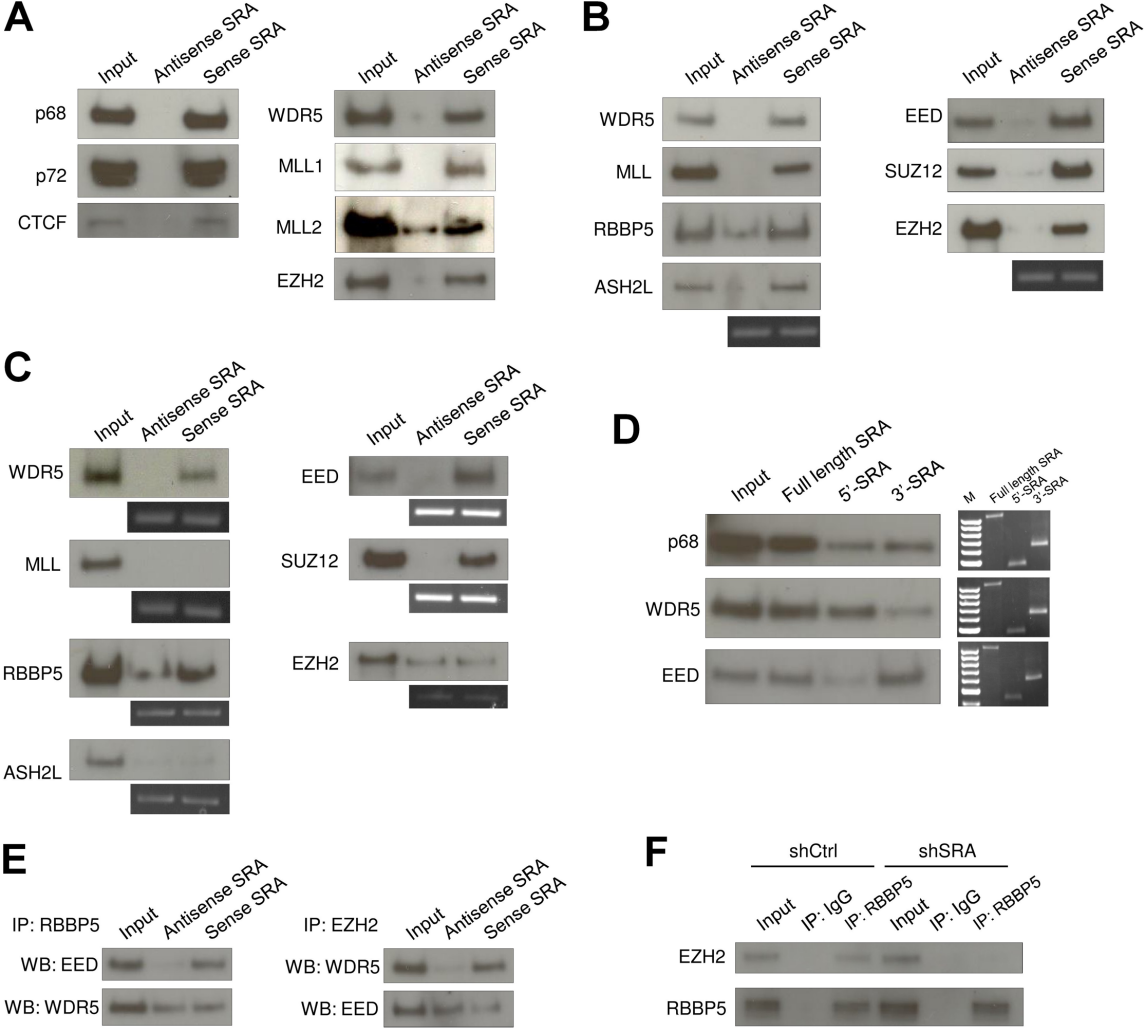
The lncRNA SRA directly interacts with TrxG and PRC2 complexes. (A) RNA pull down experiment using nuclear extract of human pluripotent stem cells NTERA2 followed by western blotting of indicated proteins. (B) Purified recombinant TrxG and PRC2 complexes were used for *in vitro* RNA pull down experiment and western blotting. Below: RT-PCR of antisense or sense SRA purified from *in vitro* pull down reactions. (C) The TrxG core component WDR5 and the PRC2 component EED and SUZ12 directly associate with SRA. Purified recombinant proteins were used for RNA pull down experiment and western blotting. (D) 5’ and 3’ domains of SRA are preferential binding regions for TrxG and PRC2 complexes, respectively. Right: RT-PCR of full length, 5’ and 3’ domains of SRA from pull down reactions; these three panels represent independent experiments and should not be compared. (E) SRA can tether TrxG and PRC2 complexes. Co-immunoprecipitation (Co-IP) was performed using purified TrxG and PRC2 complexes in the presence of antisense or sense SRA. Left: Co-IP using RBBP5 antibody. Right: Co-IP using EZH2 antibody. (F) SRA mediates interaction between TrxG and PRC2 *in vivo*. Co-IP was performed by using nuclear extract of scrambled RNA control and *SRA* knockdown NTERA2 cells. All experiments were performed as at least two independent replicates. The inputs were used at 10% of the samples.

To confirm that SRA interacts with the RNA helicase p68 and CTCF [26], an RNA pull down assay was performed using nuclear extract from human pluripotent stem cells NTERA2 and *in vitro* transcribed biotinylated antisense SRA and sense SRA. Western blot analysis showed that sense SRA specifically recruits p68 and CTCF (Fig 1A and S1 Fig) supporting our previous report [26]. p72, another RNA helicase known to interact with SRA, was also pulled down by SRA. Next, to detect a possible association between SRA and TrxG and/or PRC2 in nuclear extract, the RNA pull down assay was employed to probe for WDR5 and EZH2 proteins, respectively. Both WDR5 and EZH2 were pulled down selectively by sense SRA suggesting that SRA interacts with TrxG and PRC2 complexes (Fig 1A). WDR5 is shared by several TrxG complexes: interaction with both MLL1 and MLL2 was detected in these pull down experiments (Fig 1A), as were related complexes containing histone methyltransferases SETD1A and SETD1B (S2 Fig). RNA immunoprecipitation experiment showed that SRA was retrieved by anti-WDR5 and anti-SUZ12, indicating an association between SRA and TrxG/PRC2 complexes *in vivo* (S3 Fig). An *in vitro* RNA pull down assay similarly revealed an interaction between SRA and either recombinant TrxG or PRC2 complexes indicating that the binding between SRA and the two epigenetic machineries is direct (Fig 1B). The selective properties of the SRA sense strand, in contrast to the antisense strand, are consistent with a specific interaction between the RNA and the two histone modifying complexes.

To determine which components of TrxG and PRC2 mediate the interaction with SRA, individual recombinant proteins were used in the RNA pull down. Among major TrxG components, sense SRA specifically retrieved WDR5, whereas it pulled down both the EED and SUZ12 components of the PRC2 complex (Fig 1C). This result indicates that SRA interacts with TrxG through WDR5 and with PRC2 via EED and SUZ12. Purified EZH2, when not part of the PRC2 complex, shows no selective affinity for sense as compared to anti-sense SRA (Fig 1C). To a lesser extent this is true for RBBP5, which as an isolated component shows some binding to anti-sense SRA, unlike other members of the TrxG complex (Fig 1B). It is clear however that the full complexes, and most of their components, exhibit selective binding to sense SRA.

Domain mapping analysis, in which the 5’ or 3’ halves of the SRA molecule are separately tested for their ability to interact with TrxG and PRC2 complexes, suggests that the TrxG and PRC2 complexes preferentially bind to the 5’ and 3’ regions of SRA, respectively (Fig 1D and S4 Fig). We note that the secondary structure of SRA [30] harbors distinct domains that might be specialized to interact with the TrxG and PRC2 complexes. These observations raise the question whether SRA might simultaneously bind to both TrxG and PRC2, thereby in principle allowing for delivery of both activating and silencing marks. Co-immunoprecipitation experiments were performed using recombinant TrxG and PRC2 complexes in the presence of either antisense or sense SRA. Immunoprecipitation of RBBP5 resulted in an enrichment of EED when sense SRA was present in the reaction (Fig 1E). Similarly, immunoprecipitation of EZH2 led to an enrichment of WDR5 in the presence of sense, but not antisense SRA. These results indicate that TrxG, PRC2 and SRA are present in the same complex. However they do not distinguish between a complex in which a single SRA molecule binds both TrxG and PRC2, and, for example, a complex containing two or more SRA molecules, each separately carrying either TrxG or PRC2. Nonetheless, the experiment in Fig 1D suggests that the binding domains on SRA for each complex are largely independent of each other and should be capable of binding both complexes at once

To determine whether SRA displays the same bi-faceted binding properties *in vivo*, shRNA silencing of *SRA* was employed to deplete *SRA* expression in NTERA2 (S5 Fig). Immunoprecipitation of RBBP5 co-precipitated EZH2 in control knockdown cells (Fig 1F). However, this interaction of EZH2 and RBBP5 was reduced in *SRA* knockdown cells. This result is consistent with the *in vitro* interaction assay and suggests that SRA may be capable of delivering both activating and silencing histone modifications to sites where it is bound.

### p68 facilitates SRA and TrxG interaction

**Fig 2:**
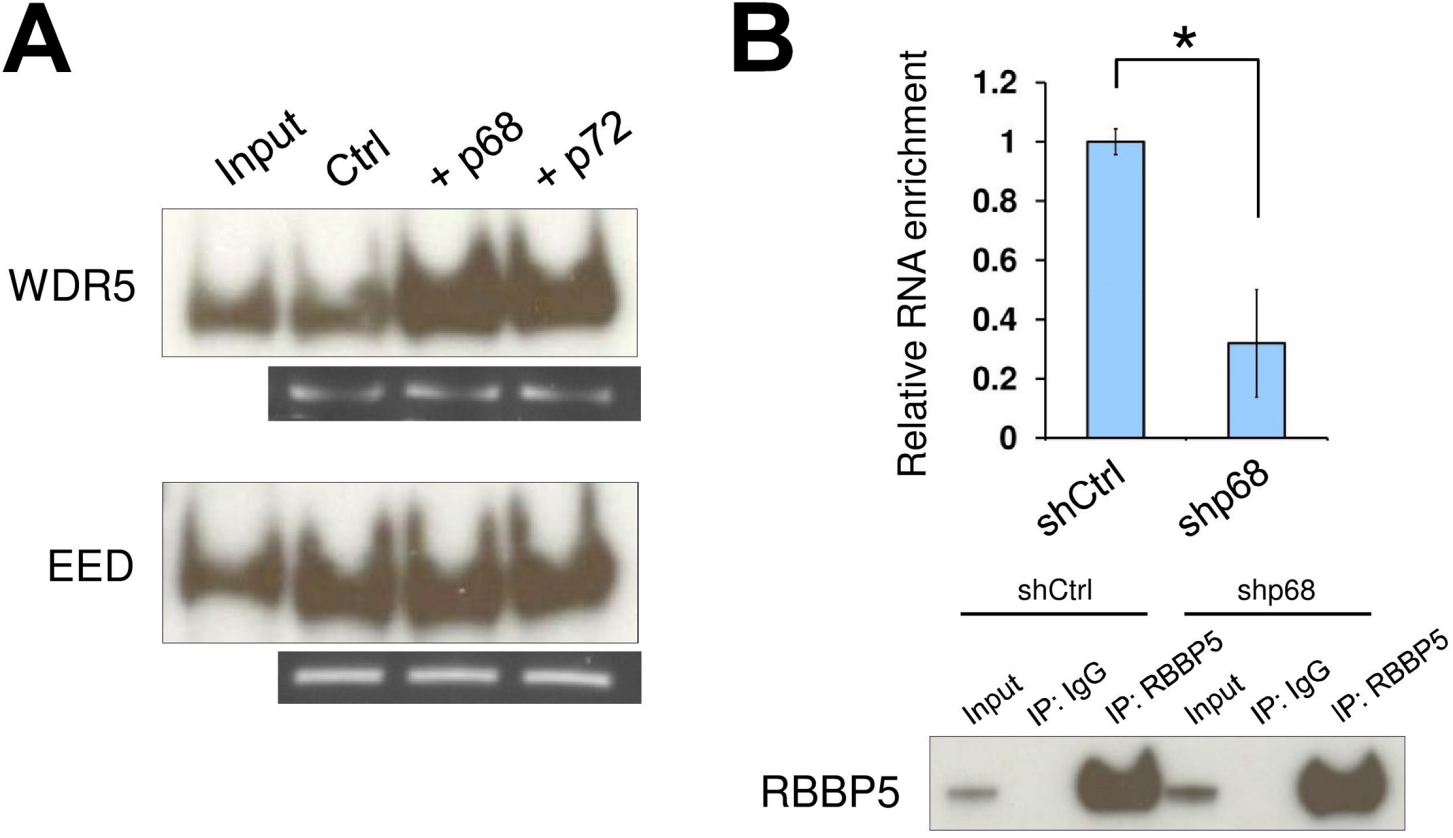
The RNA helicase p68 facilitates TrxG recruitment by SRA. (A) Interaction between TrxG complex and SRA is enhanced by either p68 or its highly conserved homologue p72. Purified recombinant TrxG and PRC2 complexes were used for *in vitro* RNA pull down experiments in the absence or presence of recombinant p68 and p72. (B) p68 promotes interaction between TrxG complex and SRA *in vivo*. RNA immunoprecipitation was performed using scramble and p68 knockdown NTERA2 cells. Upper: qPCR of SRA purified from immunoprecipitates using anti-rabbit RBBP5 antibody. Data are shown as mean±SD; n = 3. * *p* < 0.05. *p* value calculated with two-tailed Student’s *t* test. Lower: Western blot of immunoprecipitates using anti-mouse RBBP5 antibody. The inputs were used at 10% of the samples.

The lncRNA SRA and RNA DEAD box helicase p68 have been implicated as acting together in transcriptional regulation, yet their mechanism of action remains elusive. If SRA in the absence of other components can recruit both the TrxG and PRC2 complexes, what role does p68 play? We therefore sought to establish whether p68 might modulate SRA/TrxG/PRC2 interactions, altering the affinity of SRA for these complexes. An SRA pull down assay shows that the amount of interacting TrxG complexes is increased when p68 is present in the reaction (Fig 2A). This property of p68 to promote TrxG recruitment by SRA is not due to an interaction between p68 and TrxG, since p68 does not directly associate with TrxG complexes (S6 Fig). In contrast, the ability of SRA to pull down PRC2 complex is not altered by p68 (Fig 2A). We obtained similar results using the p68 homolog p72. To confirm *in vivo* the function of p68 in promoting SRA and TrxG interaction, RNA immunoprecipitation was carried out with an antibody recognizing RBBP5 after using shRNA to knock down p68 (S7 Fig). The result shows that enrichment of SRA bound to TrxG complex, but not to PRC2, was reduced in p68 knockdown cells (Figs 2B and S8). These results thus reveal a role of p68 in facilitating interaction between the lncRNA SRA and the activating epigenetic machinery of the TrxG complex.

### SRA co-localizes with bivalent domains genome-wide

**Fig 3:**
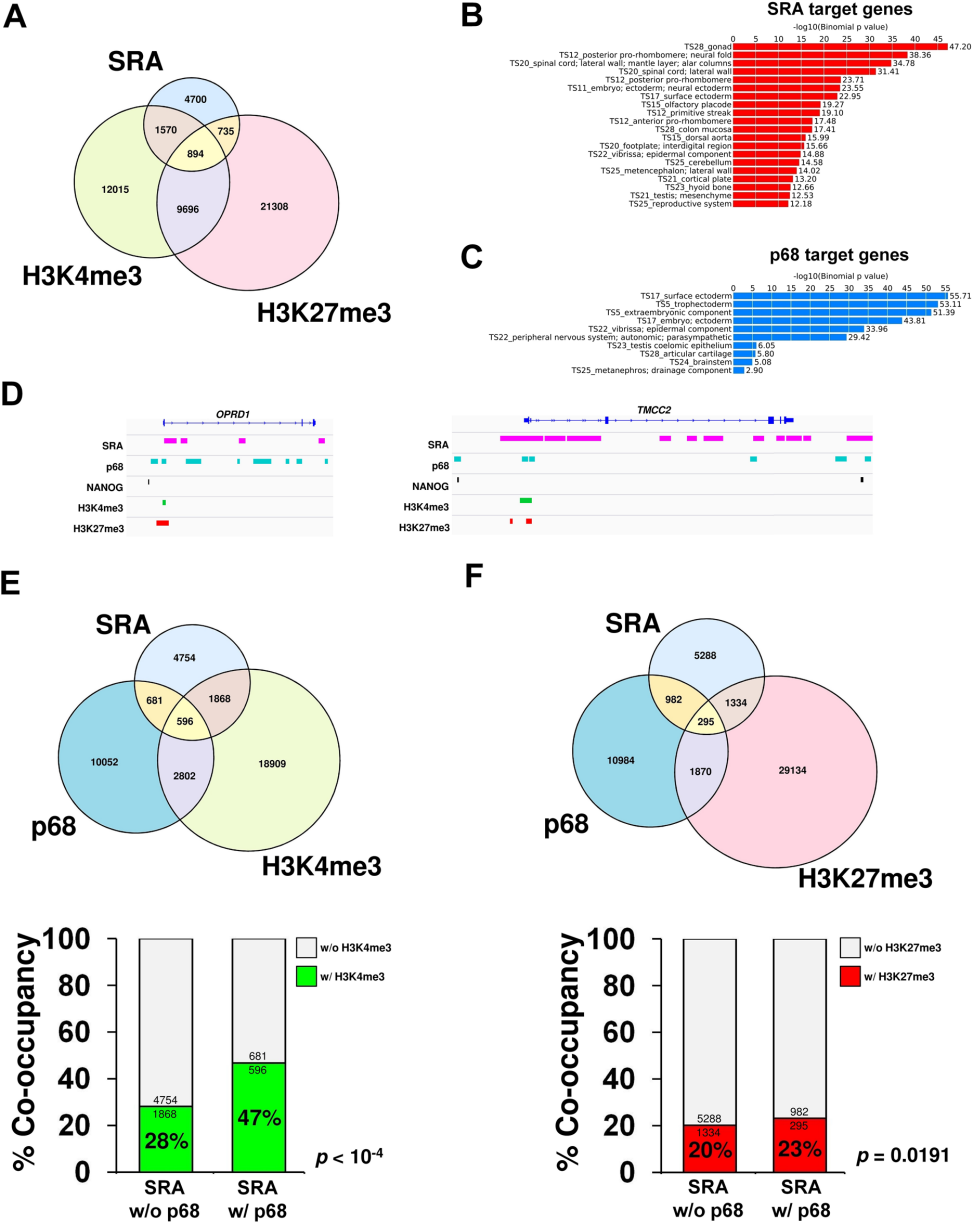
p68 and SRA colocalize at bivalent promoters in pluripotent stem cells. (A) Venn diagram of regions bound by SRA, H3K4me3 and H3K27me3 in human pluripotent stem cells NTERA2. ChIRP-seq analysis of SRA was performed, and the resulting SRA-binding sites were compared with regions in NTERA2 occupied by H3K4me3 and H3K27me3 from the ENCODE project. *Statistical analysis of the association between SRA*, *H3K4me3 and H3K27me3 using Fisher’s exact test*. *shows that the association of SRA with the two histone marks is statistically significant with p*-value < 10^−4^. (B and C) Categories of SRA- and p68-associated genes, respectively, were analyzed using GREAT based on MGI Expression. (D) Examples of ChIRP-seq and ChIP-seq gene tracks showing occupancy of SRA and p68 on two genes marked by bivalent modification, which are also associated with NANOG. Publicly available data for H3K4me3, H3K27me3 and NANOG ChIP-seq were derived from the ENCODE project. (E and F) Genome-wide p68-binding sites were compared with SRA and H3K4me3 (E) or H3K27me3 (F). Lower; Percentage of co-occupancy of H3K4me3 (E) and H3K27me3 (F) at SRA binding sites without or with p68 occupancy. *p* values were calculated by Fisher’s exact test.

SRA interacts directly with TrxG and PRC2 complexes. The function of PRC2 involves methylation of histone H3 lysine 27. The TrxG complex carrying MLL2 is responsible for trimethylation of histone H3 lysine 4 in mouse embryonic stem cells [1, 2], particularly at bivalent sites. We therefore asked whether SRA might be present at bivalent domains. To this end, we utilized the ChIRP technique [19] to pull down the lncRNA SRA from chromatin of the human pluripotent stem cells NTERA2. Using next generation sequencing, we identified 7,899 SRA-binding sites genome-wide (see Methods). Comparing SRA with profiles of H3K4me3 and H3K27me3 in NTERA2 generated by the ENCODE project, we find that 1,570 and 735 sites representing 20% and 9.3% of total SRA binding sites possess respectively either the H3K4me3 or H3K27me3 modification exclusively (Fig 3A). Among SRA binding sites, 894 regions representing 11% have the bivalent domain signature (Fig 3A, 3D and S9 Fig). Taken together, about 40% of SRA sites carry at least one of these modifications. Of all bivalent domains we mapped, 8% are associated with SRA binding. Gene classification analysis reveals that SRA-bound regions are associated with differentiation and embryonic development genes (Fig 3B). This result is consistent with the observed interaction *in vitro* and *in vivo* between SRA and TrxG/PRC2 complexes, and with a role for SRA in targeting histone modifications, including bivalent modifications, in pluripotent stem cells.

Because p68 facilitates interaction between SRA and WDR5 containing complexes, we asked whether sites of H3K4me3 modification might be enriched at genomic regions occupied by both SRA and p68 relative to those occupied by SRA alone. Chromatin immunoprecipitation (ChIP) sequencing of p68 in NTERA2 identified 14,131 binding sites genome wide; functions of many associated genes are involved in embryonic development (Fig 3C). It is obvious from our data that many sites of H3K4 or H3K27 methylation are associated neither with p68 nor SRA, consistent with the existence of multiple mechanisms for delivering those modifications. However if we focus on the role of SRA and its interaction with p68, we find that 16% of SRA binding sites are also occupied by p68 (Fig 3D–3F and S10 Fig). Furthermore 21% of SRA/p68 binding sites are located at bivalent sites that harbor both H3K4me3 and H3K27me3 marks (S11 Fig). Interestingly, we observe a significant 19% (47% versus 28%) increase (*p*-value < 10^−4^, Fisher’s exact test) in sites carrying the H3K4me3 modification at genomic regions occupied by both SRA and p68 compared with those occupied by SRA but lacking p68 (Fig 3E). To investigate whether p68 facilitates modification of H3K4me3, we performed ChIP-PCR of the histone mark at selected p68-bound genes upon silencing of p68. Depletion of p68 led to a decrease in H3K4me3 occupancy at half of the p68-bound genes we examined (S12 Fig). On the other hand, the presence of p68 at SRA binding sites has an insignificant effect on the extent of H3K27me3 modification (23% versus 20%) (Fig 3F). The genome-wide accumulation of H3K4me3 at p68-associated SRA binding sites thus suggests a role *in vivo* for p68 in facilitating SRA mediated H3K4 methylation, consistent with our observations *in vitro* that p68 stabilizes SRA-TrxG interaction.

### SRA interacts with the pluripotency-associated transcription factor NANOG

We have previously shown that CTCF, a DNA binding protein, interacts with p68/SRA in nuclear extracts, and that p68 binding is essential to CTCF dependent insulator function at the human *IGF2/H19* imprinted locus [26]. However unlike the interactions of SRA with TrxG or PRC2, the interaction between CTCF and p68/SRA is indirect (S13 Fig). Analysis of SRA ChIRP data from the pluripotent stem cells NTERA2 cells shows that not all CTCF sites are associated with SRA. Nonetheless, recruitment of SRA by CTCF increases the probability that the site will also be bivalent: 14.3% of sites occupied by both CTCF and SRA also carry bivalent marks, whereas only 7.3% of CTCF sites not associated with SRA are bivalent (S14 Fig, *p*-value < 10^−4^). The presence of SRA at CTCF binding sites thus correlates with the presence of bivalent domains.

**Fig 4:**
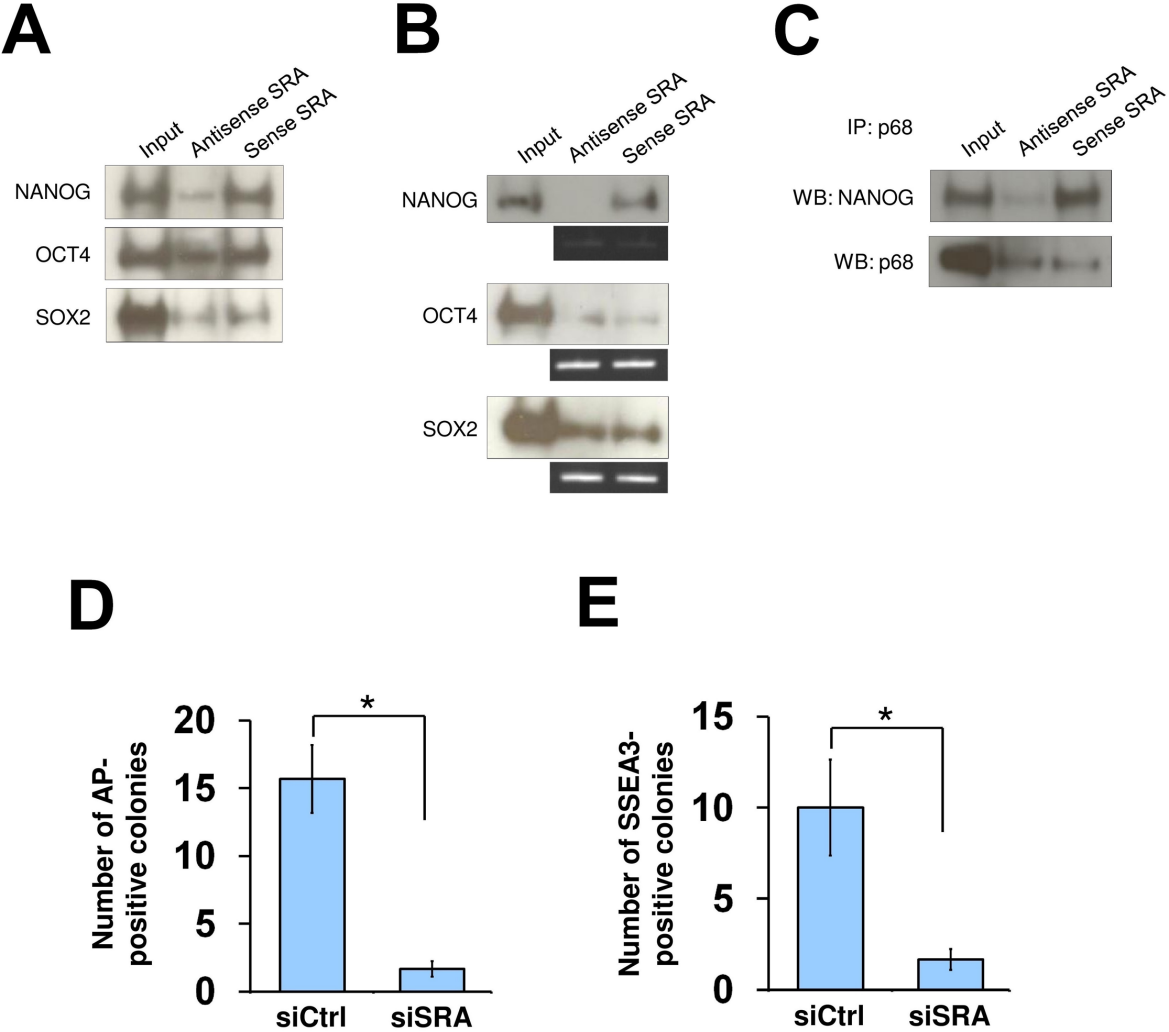
SRA directly interacts with NANOG and is important for reprogramming of iPS cells. (A) RNA pull down using nuclear extract of human pluripotent stem cells NTERA2, followed by western blot analysis of indicated proteins. (B) NANOG interacts with sense SRA. Recombinant proteins were used for RNA pull down experiment and western blotting. (C) SRA forms a complex with NANOG and p68. Co-IP was performed using recombinant NANOG and p68 in the presence of antisense or sense SRA. All experiments were performed as at least two independent replicates. The inputs were used at 10% of the samples. (D and E) SRA facilitates reprogramming of iPS cells. Human fibroblasts were transfected with a plasmid over-expressing the reprogramming factors OCT4, SOX2, c-MYC and KLF4 without or with *SRA* silencing. At day 30 cells were stained for alkaline phosphatase (AP) (D) or SSEA3 (E). Data are shown as mean±SD; n = 3. * *p* < 0.05. *p* value calculated with two-tailed Student’s *t* test.

We next asked whether the core transcription factors NANOG, OCT4 and SOX2, which have been shown to occupy sites at bivalent genes in human pluripotent stem cells [31, 32], might interact with SRA as a means to recruit the lncRNA to their target genes. RNA pull down experiments using either nuclear extract or recombinant proteins reveal a direct association between SRA and NANOG, but our data do provide evidence for such association of OCT4 or SOX2 (Fig 4A and 4B). Further, co-immunoprecipitation of p68 and NANOG in the presence of sense or antisense SRA shows that SRA facilitates specific complex formation between p68 and NANOG (Fig 4C). Using a publicly available ENCODE database of NANOG ChIP-seq in human embryonic stem cells, we find that 16% of SRA binding sites detected in NTERA2 cells overlap with NANOG (S15 Fig). Unlike for CTCF, we do not find a correlation between SRA co-localization with NANOG and the abundance of bivalent domains. However, 16.5% of NANOG-SRA binding sites also show bivalent association (S16 Fig). At NANOG-SRA binding sites, the H3K4me3 mark associates with 75% of regions when p68 is present (NANOG/SRA/p68/K4Me3 vs all NANOG/SRA/p68) compared with 51% of this modification at these regions without p68 (NANOG/SRA/K4Me3 no p68 vs all NANOG/SRA no p68) (S16B Fig, *p*-value < 10^−4^). In contrast, a 5% reduction of H3K27me3 co-occupancy is observed for NANOG-SRA binding sites when p68 is present (S16C Fig). Thus, similar to the above observation for all SRA associated sites, the presence of p68 at NANOG-SRA binding sites appears to facilitate the establishment of H3K4 methylation.

As the TrxG and PRC2 complexes are important for reprogramming of somatic cells toward induced pluripotent stem cells [4, 33, 34], we tested whether SRA is also important for this process. Human fibroblasts were transfected with a plasmid encoding OCT4, SOX2, c-MYC and KLF4, and were grown under feeder-free human pluripotent stem cell conditions for 30 days. We find that, when *SRA* expression is depleted, the numbers of alkaline phosphatase and SSEA3 positive colonies are reduced (Fig 4D and 4E). This observation suggests that, similar to TrxG and PRC2 complexes, SRA is a crucial factor for the reprogramming of fibroblasts toward induced pluripotent stem cells. Additionally, we find that silencing of *SRA* leads to a decrease in number of cells expressing the pluripotent stem cell marker SSEA3, while the number of cells expressing the differentiation marker A2B5 is increased (S17 Fig). This result indicates that SRA is important for maintaining the stem cell state. However, because silencing of SRA results in a decrease in self-renewal, we are unable to carry out experiments to study the effects of SRA depletion on histone modifications while maintaining the stem cell identity of NTERA2 cells.

## Discussion

The enzymatic mechanisms and cofactors underlying H3K4 and K27 trimethylation have been well characterized. However, little is known about mechanisms that could selectively generate a bivalent domain, which carries both kinds of methylation marks. In the present study, we have identified SRA as a lncRNA interacting with both the TrxG and PRC2 complexes. As discussed in the Introduction, several lncRNAs have been shown to bind either to TrxG or PRC2 [12, 13, 15–17]. To date, the only lncRNA known to interact with both TrxG and PRC2 is Fendrr [35]. However, it is not known whether the interaction between Fendrr and the two histone modifying complexes is direct, or whether Fendrr can deliver those complexes simultaneously. In NTERA2 cells, 11% of SRA-binding sites genome-wide overlap with bivalent domains, and another 29% are associated with sites carrying either H3K4me3 or H3K27me3. This suggests that, depending upon the site, SRA can deliver either or both of these modifications, in the latter case consistent with the presence of a bivalent mark.

Although SRA possesses a potential to interact with both TrxG and PRC2, 20% of SRA-binding sites are occupied by H3K4me3 but not H3K27me3, whereas only 9% of SRA-binding sites are marked by H3K27me3 but not H3K4me3. Our finding therefore supports a preferred role of SRA as a transcriptional co-activator [29]. SRA frequently functions with p68 as a complex that can in turn interact with a variety of DNA-binding transcription factors such as MyoD. But as shown here for SRA-NANOG, SRA in some cases does not require the assistance of p68. Our data nonetheless show that the presence of p68 enhances interaction between SRA and the TrxG complex in experiments carried out either with purified components or with nuclear extracts (Fig 2). The role of p68 in increasing SRA-TrxG interaction is analagous to that of ATRX, which increases interaction between Xist and PRC2 [36]. Consistent with these observations, the presence of p68 at SRA sites in NTERA2 cells *in vivo* increases the co-occupancy between SRA and H3K4me3 from 29% to 52% (Fig 3E). These findings reveal the mutual relationship between p68 and SRA in transcriptional activation.

Many DNA-binding transcription factors have been reported to interact with SRA, either directly or indirectly [22]. Our study shows that SRA directly interacts with the homeodomain transcription factor NANOG, which occupies regulatory elements of many genes associated with bivalent domains in human pluripotent stem cells [31, 32]. We find that SRA and NANOG share binding sites genome-wide. NANOG is a key transcription factor required for self-renewal of human and mouse embryonic stem cells [37–39] and for establishment of pluripotency [40]. Similar to the latter function of NANOG, TrxG and PRC2 complexes are also important for reprogramming of the pluripotent state [4, 33, 34]. Our results suggest that NANOG recruits SRA and its associated TrxG and PRC2 complexes as part of the mechanism for establishing the pluripotency of induced pluripotent stem cells, and at least in some cases plays a role in establishing and/or maintaining bivalent domains (see model in S18 Fig). Our results also show that SRA localization sites are widespread in the genome, and that they are likely to be involved at those sites in delivery of both activating and silencing histone modifications. The SRA/TrxG/PRC2 complexes can be recruited directly or indirectly to binding sites on DNA through interaction with a variety of transcription factors, only some of which have so far been identified. CTCF is a ubiquitous factor that appears to contribute to establishment of bivalent states at sites where SRA is also present. In addition to recruiting both MLL1 and MLL2, which trimethylate H3K4, SRA recruits both SETD1A and SETD1B, raising the possibility that it may mediate histone H3 monomethylation as well as trimethylation. Many other factors (such as MyoD and NANOG) are lineage specific; it will be important to investigate in other cell types the interaction of the SRA/TrxG/PRC2 complexes with lineage specific transcription factors, and their role in establishing patterns of histone modification important for regulation of gene expression.

## Materials and Methods

### Plasmid constructs

A plasmid containing SRA sequence (BC067895.1) was purchased from Open Biosystems. The SRA coding sequence was subcloned into pLITMUS28i (New England Biolabs) for *in vitro* transcription (see below). The following plasmids were used for in *vitro* transcription/translation: pSG5-MYC encoding p68 and p72 (gift from Prof. Frances V. Fuller-Pace, University of Dundee, UK); pcDNA3.1-NANOG (Addgene).

### Antibodies

See S1 Table for the list of antibodies used in this study.

### Cell culture and transfection

Human pluripotent stem cell line NTERA2 was grown in DMEM supplemented with 10% FBS (Gibco) at 37°C under a humidified atmosphere of 5% CO_2_ in air. At confluent, cells were passaged every three days using 0.25% trypsin (Gibco). For establishment of NTERA2 stable knockdown cell lines, the plasmids pMLP-shRNA targeting SRA, p68 or scramble control (transOMIC) were linearized by *Nde*I and transfected into 1x10^6^ cells using nucleofector (Amaxa) according to manufacturer’s protocol. Cells were immediately grown in DMEM-F12 plus 10% FBS. On day 3, stable cell lines were selected using puromycin at 3 μg/ml final concentration.

### RNA pull down

RNA pull down experiments were performed as previously described [41]. First, DNA fragments encoding full length, 5’ and 3’ domains of lncRNA SRA were cloned into pLITMUS28i (New England Biolabs), and the DNA sequence was confirmed by sequencing. To generate antisense or sense SRA transcripts, the plasmid containing full length SRA was linearized by *Stu*I or *Bgl*I, respectively. Biotinylated SRA was *in vitro* transcribed using HiScribe T7 *In Vitro* transcription kit (New England Biolabs) in the presence of biotin-14-CTP (Invitrogen) according to the instruction manuals. Transcribed RNA products were DNase-treated (Ambion), purified by ethanol precipitation and verified by northern blotting.

For RNA pull downs using nuclear extract, 3 μg of *in vitro* transcribed RNA was prepared in RNA structure buffer (Tris-Cl pH 7.5, 0.1 M KCl, 10 mM MgCl_2_) and incubated at 78°C for 3 min. The RNA was then gradually cooled down to 37°C. Five hundred micrograms of NTERA2 nuclear extract, prepared using NE-PER Nuclear Protein Extraction Kit (Pierce), was mixed with the RNA in immunoprecipitation buffer (PBS plus 0.1% Triton X-100, 1 mM DTT, protease inhibitor cocktail, PMSF, 80 U RNase inhibitor) in a total volume of 500 μL. The reaction was incubated for 4 hr at 4°C with rotation. MyOne Streptavidin C1 beads were prepared according to manufacturer’s recommendation, and used at 50 μL per sample. The RNA-beads complex was further incubated overnight. Beads were washed five times with immunoprecipitation buffer and boiled with 50 μL of SDS loading buffer. Twenty microliters was loaded onto Novex precast gel (Invitrogen). For RNA pull down using recombinant proteins, 0.3 μg of RNA was used per pull down reaction with 3 μg of protein complex or 1 μg of individual protein. TrxG and PRC2 complexes were purchased from BPS Bioscience and Cayman Chemical. NANOG, OCT4 and SOX2 were purchased from Fitzgerald Industries International. The RNA helicases p68 and p72 were *in vitro* translated using the TNT Coupled Reticulocyte Lysate System (Promega). A plasmid encoding luminescence protein was used as negative control (Promega). Recombinant NANOG was also produced by *in vitro* translation using a Wheat Germ System (Promega). All *in vitro* translated proteins were verified by western blotting. Ten microliters of translated protein product was used per RNA pull down reaction.

### Co-immunoprecipitation

For *in vitro* co-immunoprecipitation in the presence of antisense or sense SRA, the RNAs were transcribed without Biotin-14-CTP. Three micrograms of TrxG and PRC2 complexes or 10 μL of *in vitro* translated p68 and NANOG were used for co-immunoprecipitation in 200 μL of immunoprecipitation buffer. Antibodies for immunoprecipitation were used at 3 μg including mouse anti-RBBP5 (MABE220, Upstate), mouse anti-EZH2 (MA5-15101, Thermo Scientific) and rabbit anti-DDX5 (A300-523A, Bethyl Laboratories).

For co-immunoprecipitation using nuclear extract, 500 μg of NTERA2 nuclear extract was mixed with 3 μg of relevant antibodies in a total of 500 μL of immunoprecipitation buffer. The reaction was incubated for 4 hr at 4°C with rotation. Protein A and protein G conjugated magnetic beads were prepared according to manufacturer’s recommendation (Invitrogen), and used at 50 μL per sample. The complex was then further incubated overnight. Beads were washed five times with immunoprecipitation buffer and boiled with 50 μL of SDS loading buffer. Twenty microliters was loaded onto Novex precast gel (Invitrogen).

### RNA extraction and quantitative PCR

RNA was extracted using TRIzol reagent (Invitrogen) and DNase-treated (DNA-free kit, Ambion). Complementary DNA synthesis was performed with 1 μg RNA using a Maxima First Strand cDNA Synthesis Kit (Thermo Scientific). qPCR was carried on by using Power SYBR Green PCR Master Mix (Applied Biosystems) in a total volume of 20 μl each well with 7900HT real-time PCR system (Applied Biosystems). Gene expression was normalized by expression level of *ACTB*. Primer sequences are available upon request.

### RNA immunoprecipitation

Twenty million cells were fixed with 1% formaldehyde in PBS for 10 min at room temperature. The fixation was quenched by adding glycine at 125 mM final concentration and incubated further for 5 min. Cells were washed and collected by centrifugation at 1500 rpm for 5 min. Nuclear extract was prepared by using NE-PER Nuclear Protein Extraction Kit (Pierce). Three micrograms of antibody was added to 500 μg of the nuclear extract in immunoprecipitation buffer (PBS, 1 mM DTT, protease inhibitor cocktail, PMSF, 80 U RNase inhibitor) in a total volume of 500 μL. The complex was incubated at 4°C for 4 hr. Protein A and protein G conjugated magnetic beads were used at 50 μL per sample. The complex was then further incubated overnight. Beads were washed five times and resuspended in 100 μL proteinase K buffer (10 mM Tris-Cl pH 7.5, 100 mM NaCl, 1 mM EDTA, 0.5% SDS) with 5 μL proteinase K (New England Biolabs). Samples were incubated at 50°C for 45 min with shaking, and boiled at 95°C for 10 min. Samples were mixed with 500 μL Qiazol by vigorous vortexing, and were incubated at room temperature for 10 min. RNA extraction was then performed using miRNeasy mini kit (Qiagen). qPCR was employed to detect RNA binding.

### Chromatin immunoprecipitation

ChIP was performed according to the manufacturer’s instruction (Active Motif). Briefly, 2 x 10^7^ cells were fixed with 1% formaldehyde in PBS for 10 min at room temperature. The fixation was then quenched by adding glycine. Cells were washed and collected by centrifugation at 1500 rpm for 5 min. Nuclei were sonicated twice using Bioruptor (Diagenode) at maximum power, 30 sec ON and 30 sec OFF for 7.5 min to obtain chromatin fragments ranging from 200–1000 bp. Fifty micrograms of sheared chromatin was used per IP with 3 μg antibody. Retrieved DNA fragments were purified by QIAquick PCR Purification Kit (Qiagen) or ethanol precipitation. Primer sequences for ChIP are listed in S2 Table.

### Chromatin isolation by RNA purification (ChIRP)

ChIRP analysis was performed according to published protocols with minor modifications based on ChIRP and Capture Hybridization Analysis of RNA Targets (CHART) techniques [19, 42, 43]. Briefly, 3x10^7^ cells were fixed with 1% glutaraldehyde for 10 min at room temperature with shaking. The fixation was stopped by adding glycine. Crosslinked cells were washed with PBS, and resuspended in 1 ml swelling buffer (25 mM HEPES pH 7.3, 10 mM KCl, 0.1% NP-40, 1 mM DTT, PMSF). Samples were incubated at 4°C for 30 min with shaking, and were collected by centrifugation. The pellet was resuspended with 350 μL of ChIRP lysis buffer, and was sonicated using Bioruptor (Diagenode) at maximum power, 30 sec ON and 30 sec OFF for 7.5 min of 6 cycles to obtain chromatin fragments ranging from 100–1000 bp. Sheared chromatin was then collected by centrifugation. Two hundred micrograms of sheared chromatin sample was pre-cleared for 1 hour using 100 μL of Ultralink-streptavidin beads (Pierce) at room temperature with shaking. The sample was then centrifuged, and supernatant was collected. The pre-cleared chromatin was used per hybridization reaction with 10 μL of 100 μM pooled 3’ Biotin TEG oligonucleotide probes (Integrated DNA Technologies). SRA probes were designed to cover SRA transcript at nucleotide position 124 to 1473 (accession number NR_045587.1) (See S3 Table for the probe sequences). LacZ probes were employed as negative control [19]. The sample and the probes were hybridized at 37°C for 4 hours with shaking. Once the hybridization was completed, 100 μL of C-1 magnetic beads (Invitrogen) was mixed with the sample to pull down the biotinylated probes. DNA was eluted in the presence of 12.5 mM D-Biotin (Invitrogen). DNA was ethanol precipitated and subjected to library preparation.

### Library preparation for ChIP sequencing

Library preparation was performed using TruSeq ChIP Sample Preparation Kit (Illumina) or MicroPlex Library Preparation Kit (Diagenode) according to manufacturer’s instruction. Three biological triplicates were used for ChIRP-seq and ChIP-seq. Briefly, 5–10 ng of DNA starting material, which was quantified by Qubit (Invitrogen), was used for each biological sample. The DNA was end-repaired, 3’ adenylated, and ligated with adapters. Then the ligated DNA was size-selected to obtain DNA fragments at 250–300 bp by agarose gel electrophoresis. The purified DNA was amplified to enrich the library. The final PCR product was purified by Agencourt AMPure XP beads (Beckman Coulter) and was submitted to the NIDDK Genomic Core Facility for high-throughput sequencing using Illumina HiSeq2500. The sequencing was performed with the run type of single-end, 50 bp read. Data were aligned against the human genome version human_hg19, and were exported into BAM file format.

### Data analysis

Aligned reads of SRA ChIRP-seq and p68 ChIP-seq were filtered with SAMTools program to remove duplicates and tags with a map quality score less than 20 [44]. MACS version 1.4.2 was used for peak calling with a threshold of *p-*value less than 10^−5^ for p68 and 10^−4^ for SRA [45]. The resulting BED files from each of biological triplicate samples were intersected using the Bioconductor package ChIPpeakAnno [46]. For SRA ChIRP-seq analysis, the SRA probe dataset was further subtracted against a LacZ probe dataset using bedtools [47]. The dataset was further screened for possible homologies with the RNA probes used in the analysis. No significant number of sequences was found with zero, one or two mismatches. Intersection between any of SRA, p68, NANOG and CTCF was performed with maximum distance of 500 bp between peaks. For the binding comparison between the above factors and H3K4me3 and H3K27me3, a distance less than 2 kb was allowed. BED files were visualized and exported using IGV [48]. H3K4me3 and H3K27me3 ChIP-seq data were taken from the ENCODE project of NTERA2 cells. NANOG and CTCF ChIP-seq data were taken from the ENCODE project of human embryonic stem cells H1. Gene classification analysis was performed using GREAT [49]. The Fishers Exact test to measure peak enrichment was taken from the Fisher’s exact function from the R package for statistical computing [50]. SRA ChIRP- and p68 ChIP-Sequencing data were submitted to GEO Datasets under accession number GSE58641.

### Induction of pluripotent stem cells

Human fibroblast cell line WI-38 at 1x10^6^ cells were transfected with a single plasmid encoding the four reprogramming factors OCT4, SOX2, c-MYC and KLF4 [51] using Nucleofector with scrambled siRNA or ON-TARGETplus siRNA targeting *SRA* (Dharmacon). Transfected fibroblasts were plated in six-well plates under Essential 8 medium (Invitrogen). The siRNA knockdown was also performed consecutively at first and second week post-transfection using Lipofectamine RNAiMAX Reagent (Invitrogen). Plates were collected on day 30 and were analyzed for expression of surface markers of human pluripotent stem cells. Alkaline phosphatase staining was performed using Alkaline Phosphatase Detection Kit (Millipore) per manufacturer’s instruction.

### Immunofluorescence staining

Cells were fixed with 4% PFA and were incubated with a monoclonal antibody against SSEA3 (gift from Prof. Peter W. Andrews, University of Sheffield, UK). A goat anti-mouse secondary antibody (IgG+IgM) conjugated with FITC (Cayman Chemical) was then used for visualization under a fluorescence microscope (EVOS).

### Flow cytometry

Single cells were collected by trypsinization, and resuspended with 10% FBS in PBS buffer. Primary antibodies SSEA3 and A2B5 (1 in 10 dilution) were added into cell suspensions containing 1x10^5^ cells in 100 μL volume. P3X was used as a negative control antibody. The reaction was incubated on-ice for 30 min, washed with PBS and resuspended with 10% FBS in PBS. One μL of FITC-conjugated Goat anti-Mouse antibody (Cayman Chemical) was added to the reaction. The reaction was incubated on-ice for another 30 min. Flow cytometric analysis was performed using Cytomic FC500 (Beckman Coulter).

## Supporting Information

S1 Fig (A) RNA products of *in vitro* transcription of antisense and sense SRA were run on formaldehyde agarose gel followed by ethidium bromide staining. (B) Northern blot analysis of antisense and sense biotinylated SRA was performed to verify biotinylation of the RNA molecules. (JPG)

S2 Fig RNA pull down experiment using nuclear extract of human pluripotent stem cells NTERA2 followed by western blotting of SETDB1A and SETDB1B. The inputs were used at 10% of the samples.
(JPG)

S3 Fig SRA associates with TrxG and PRC2 complexes *in vivo*. RNA immunoprecipitation was performed using nuclear extract of NTERA2 cells. Upper: qPCR of SRA purified from immunoprecipitates using anti-rabbit WDR5 or SUZ12 antibodies. GAPDH was served as a negative control. Data are shown as mean±SD; n = 3. * *p* < 0.05. *p* value calculated with two-tailed Student’s *t* test. Lower: Western blot of immunoprecipitates using anti-mouse RBBP5 or EZH2 antibodies. The inputs were used at 10% of the samples.
(JPG)

S4 Fig (A) Schematic representation of SRA full length, 5’ and 3’ domains. STR5 and STR7 are two known functional domains of SRA (Colley and Leedman 2011). (B) RNA products of *in vitro* transcription of full length, 5’ and 3’ domains of SRA were run on a formaldehyde agarose gel followed by ethidium bromide staining. (C) Northern blot analysis of full length, 5’ and 3’ domains of biotinylated SRA was performed to verify biotinylation of the RNA molecules. (JPG)

S5 Fig RT- qPCR analysis of SRA expression in SRA knockdown cells. The human pluripotent stem cells NTERA2 were transfected with a plasmid encoding shRNA targeting SRA. Cells stably expressing the shRNA were established by puromycin selection. Data are shown as mean± SD; n = 3.
(JPG)

S6 Fig p68 does not directly interact with TrxG complex. Purified recombinant TrxG complex was used for *in vitro* co- immunoprecipitation assay with p68. A rabbit polyclonal antibody recognizing p68 was used to pull down the RNA helicase. Western blot analysis was performed to detect p68 and TrxG interaction. The inputs were used at 10% of the samples.
(JPG)

S7 Fig Western blot analysis of p68 expression in p68 knockdown cells. The human pluripotent stem cells NTERA2 were transfected with a plasmid encoding shRNA targeting p68. Cells stably expressing the shRNA were established by puromycin selection.
(JPG)

S8 Fig p68 does not affect interaction between PRC2 complex and SRA *in vivo*. RNA immunoprecipitation was performed using scramble and p68 knockdown NTERA2 cells. Upper: qPCR of SRA purified from immunoprecipitates using anti-rabbit SUZ12 antibody. Data are shown as mean±SD; n = 3. * *p* < 0.05. *p* value calculated with two-tailed Student’s *t* test. Lower: Western blot of immunoprecipitates using anti-mouse EZH2 antibody. The inputs were used at 10% of the samples.
(JPG)

S9 Fig ChIRP-PCR of examples of SRA-associated bivalent genes in NTERA2 cells. (JPG)

S10 Fig Examples of raw tracks of SRA ChIRP-seq and p68 ChIP-seq data showing occupancy of SRA and p68 on four genes marked by bivalent modification, which are also associated with NANOG. Publicly available data for H3K4me3, H3K27me3 and NANOG ChIP-seq were derived from the ENCODE project.
(TIF)

S11 Fig (A) Venn diagram of regions bound by NANOG/p68, H3K4me3 and H3K27me3. (B) ChIP-PCR of examples of p68-associated bivalent genes in NTERA2 which are also bound by SRA. (JPG)

S12 Fig ChIP-PCR of H3K4me3 at examples of p68-associated bivalent genes in NTERA2 upon p68 knockdown. Silencing of p68 led to a decrease in H3K4me3 occupancy at a number of selected p68/bivalent target genes.
(JPG)

S13 Fig (A) SRA interacts with p68 but not CTCF. RNA pull down experiment using recombinant CTCF or p68 produced by *in vitro* translation. (B) SRA/p68 complex does not directly associate with CTCF. Co-immunoprecipitation (Co-IP) was performed using recombinant CTCF and p68 in the presence of antisense or sense SRA. Left: Co—IP using p68 antibody. Right: Co- IP using CTCF antibody. Note that in a previous publication (Yao et al. 20 10), although recombinant p68 and CTCF were used, the interactions were carried out in the presence of nuclear extracts. The inputs were used at 10% of the samples.
(JPG)

S14 Fig Venn diagram of regions bound by SRA, CTCF and bivalent sites (H3K4me3 and H3K27me3). Lower; Percentage of co- occupancy of bivalent sites of CTCF binding regions without or with SRA occupancy (see text). *p*-value was calculated by Fisher’s exact test.
(JPG)

S15 Fig Venn diagram of regions bound by SRA, NANOG and CTCF. (JPG)

S16 Fig (A) Venn diagram of regions bound by NANOG/SRA, H3K4me3 and H3K27me3. (B and C) Genome—wide p68-binding sites were compared with SRA and H3K4me3 (B) or H3K27me3 (C). Lower; Percentage of co-occupancy of H3K4me3 (B) and H3K27me3 (C) of SRA binding sites without or with p68 occupancy. *p*-values were calculated by Fisher’s exact test.
(JPG)

S17 Fig Flow cytometry analysis of cells expressing SSEA3 or A2B5 in human pluripotent stem cells NTERA2 with SRA knockdown. Data are shown as mean± SD; n = 3.
(JPG)

S18 Fig A proposed model of function of SRA as a bivalent long non-coding RNA. At bivalent genes harboring both H3K4me3 and H3K27me3 marks, SRA directly interacts with both TrxG and PRC2 complexes, and is recruited to target genes by transcription factors such as NANOG. The presence of p68 facilitates TrxG recruitment by SRA, which may in turn increase the level of H3K4me3.
(JPG)

S1 Table List of antibodies used in this study. (DOC)

S2 Table Primer sequences for ChIP-PCR and ChIRP-PCR. (DOCX)

S3 Table Sequences of ChIRP probes for SRA. (DOCX)
