# Supplementary figures and images for: Association of the Long Non-coding RNA Steroid Receptor RNA Activator (SRA) with TrxG and PRC2 Complexes
Source: PLoS Genet. 2015 Oct 23;11(10):e1005615. doi: 10.1371/journal.pgen.1005615 (PMC4619771; doi:10.1371/journal.pgen.1005615)

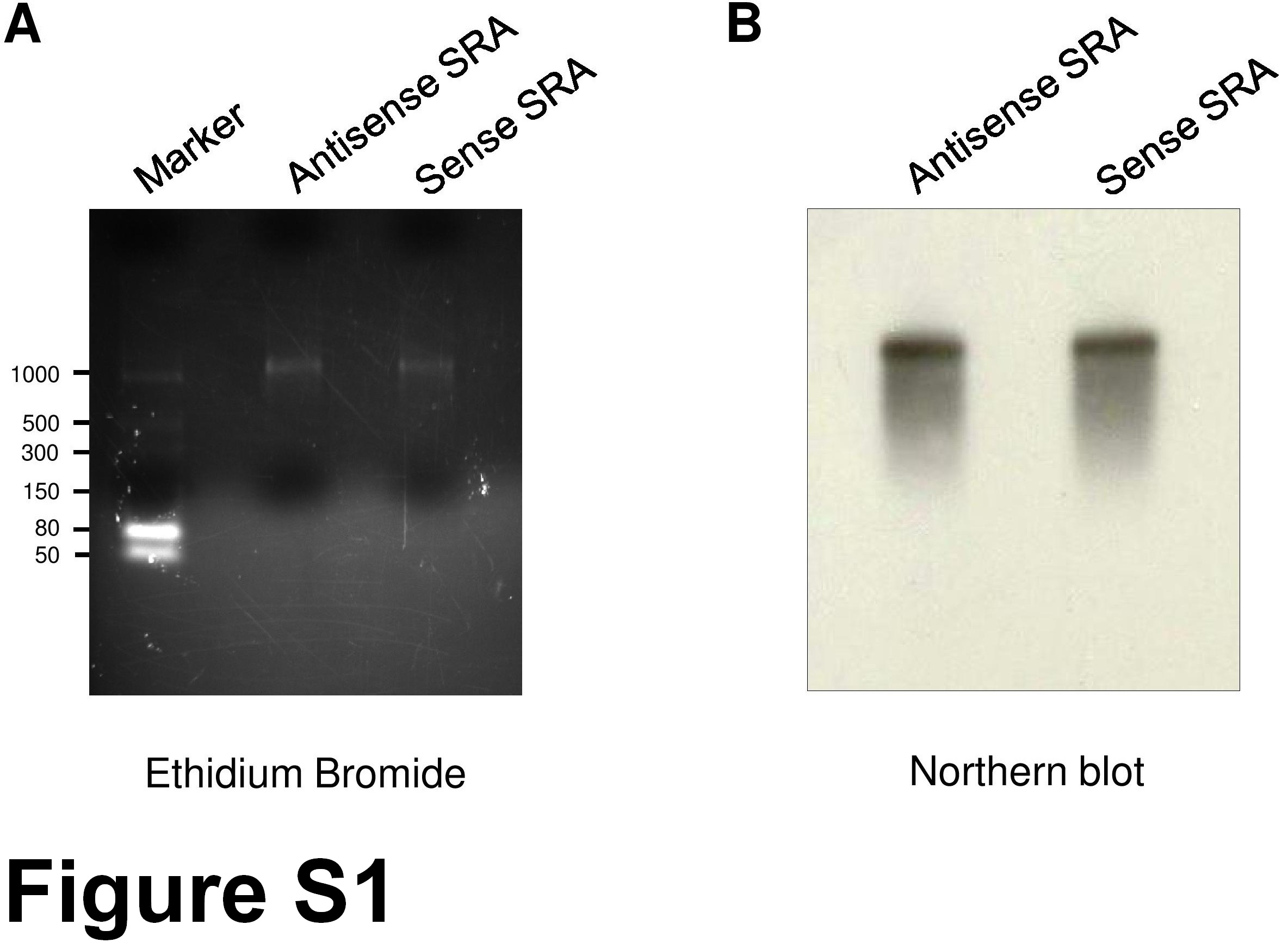

Supplement: S1 Fig — (JPG) [file pgen.1005615.s001.jpg]

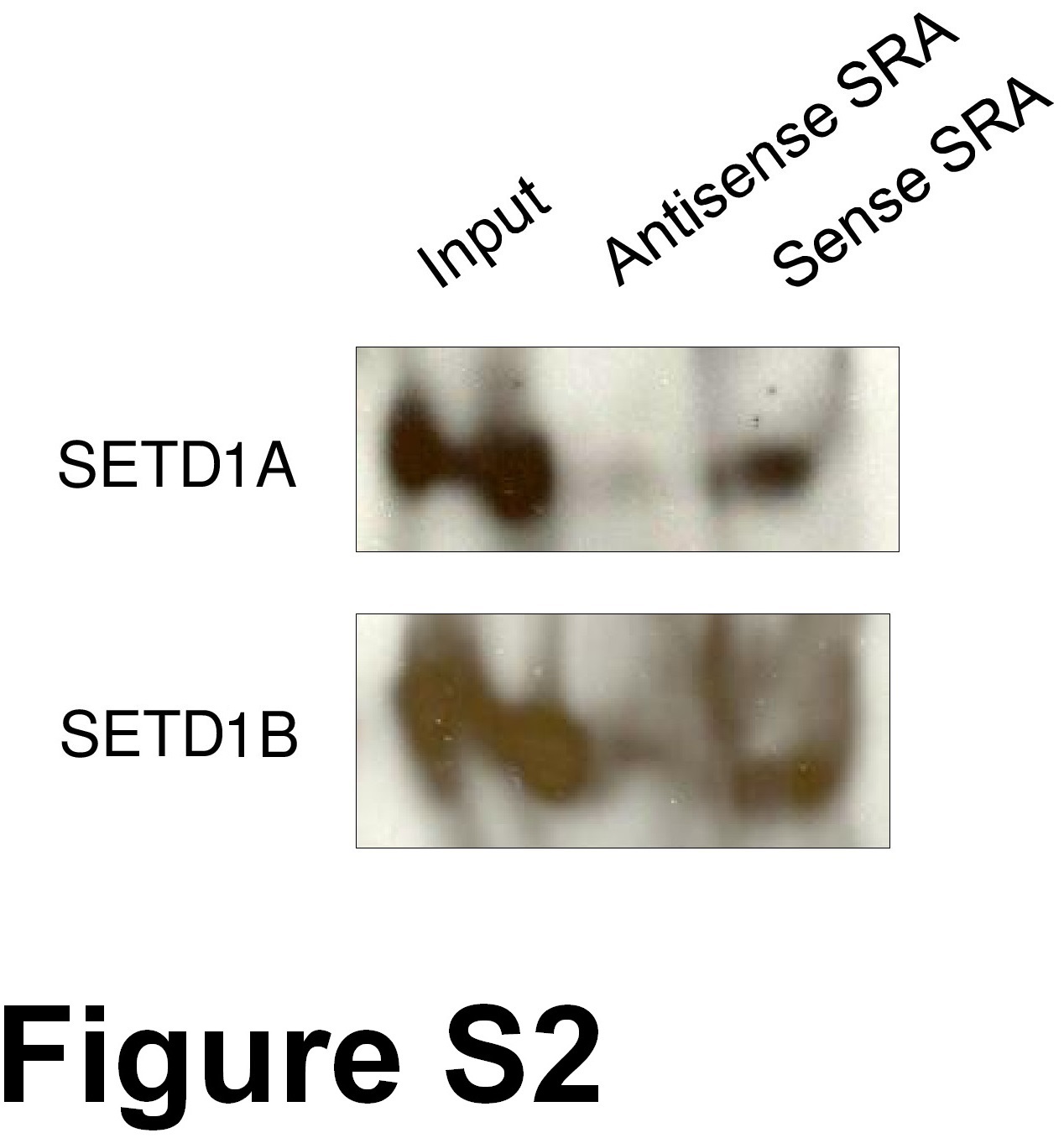

Supplement: S2 Fig — The inputs were used at 10% of the samples. (JPG) [file pgen.1005615.s002.jpg]

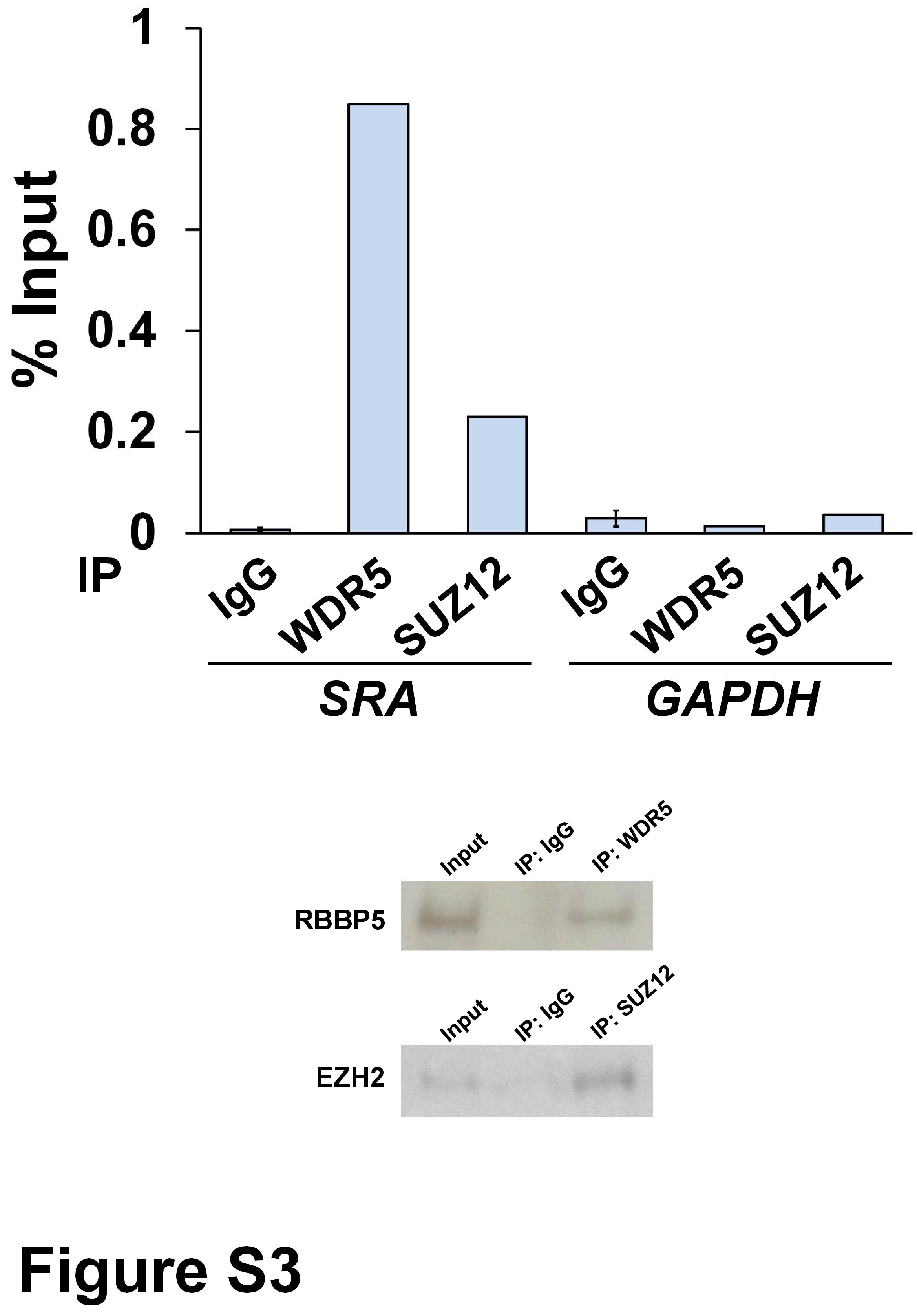

Supplement: S3 Fig — RNA immunoprecipitation was performed using nuclear extract of NTERA2 cells. Upper: qPCR of SRA purified from immunoprecipitates using anti-rabbit WDR5 or SUZ12 antibodies. GAPDH was served as a negative control. Data are shown as mean±SD; n = 3. * p < 0.05. p value calculated with two-tailed Student’s t test. Lower: Western blot of immunoprecipitates using anti-mouse RBBP5 or EZH2 antibodies. The inputs were used at 10% of the samples. (JPG) [file pgen.1005615.s003.jpg]

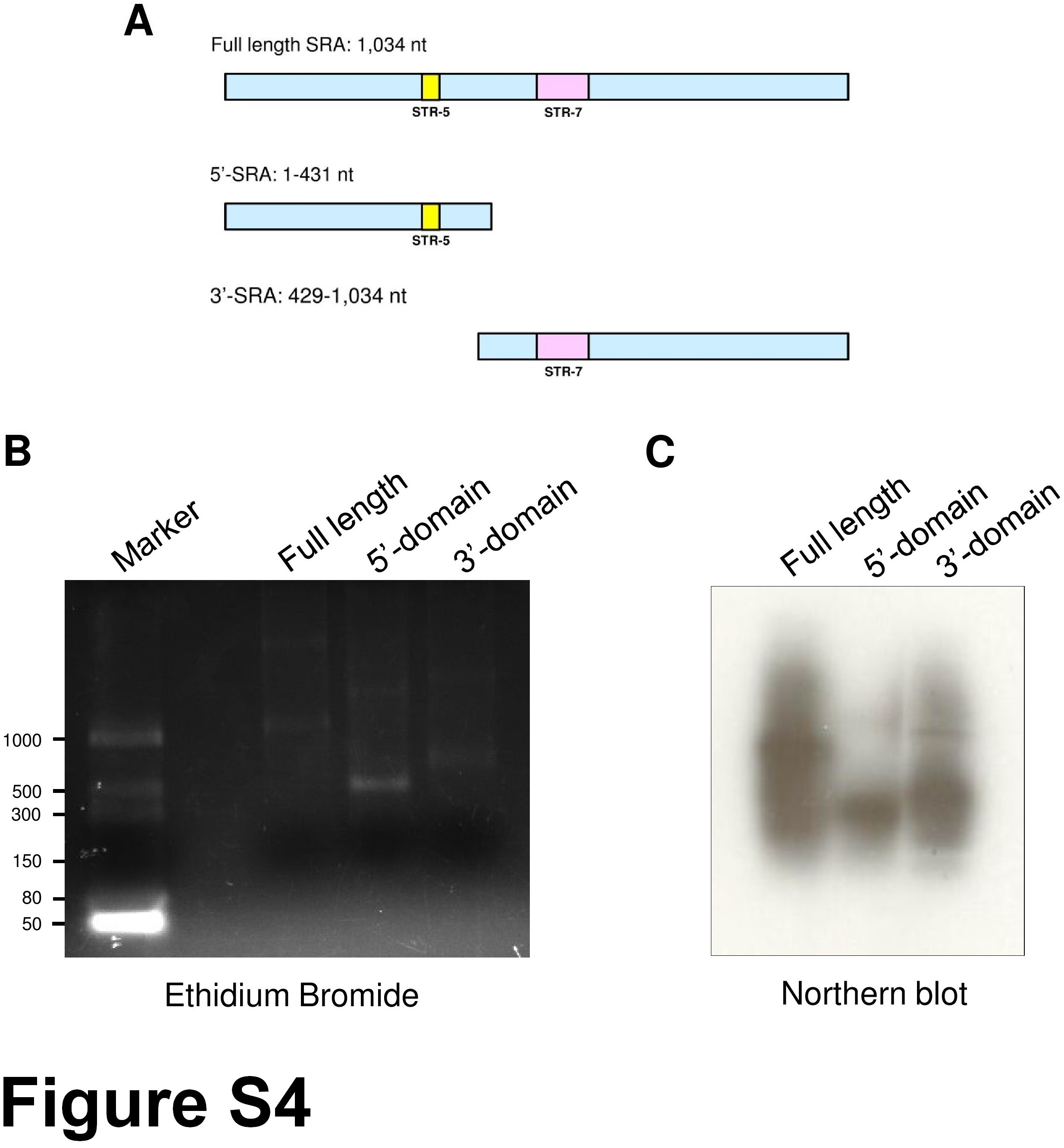

Supplement: S4 Fig — (JPG) [file pgen.1005615.s004.jpg]

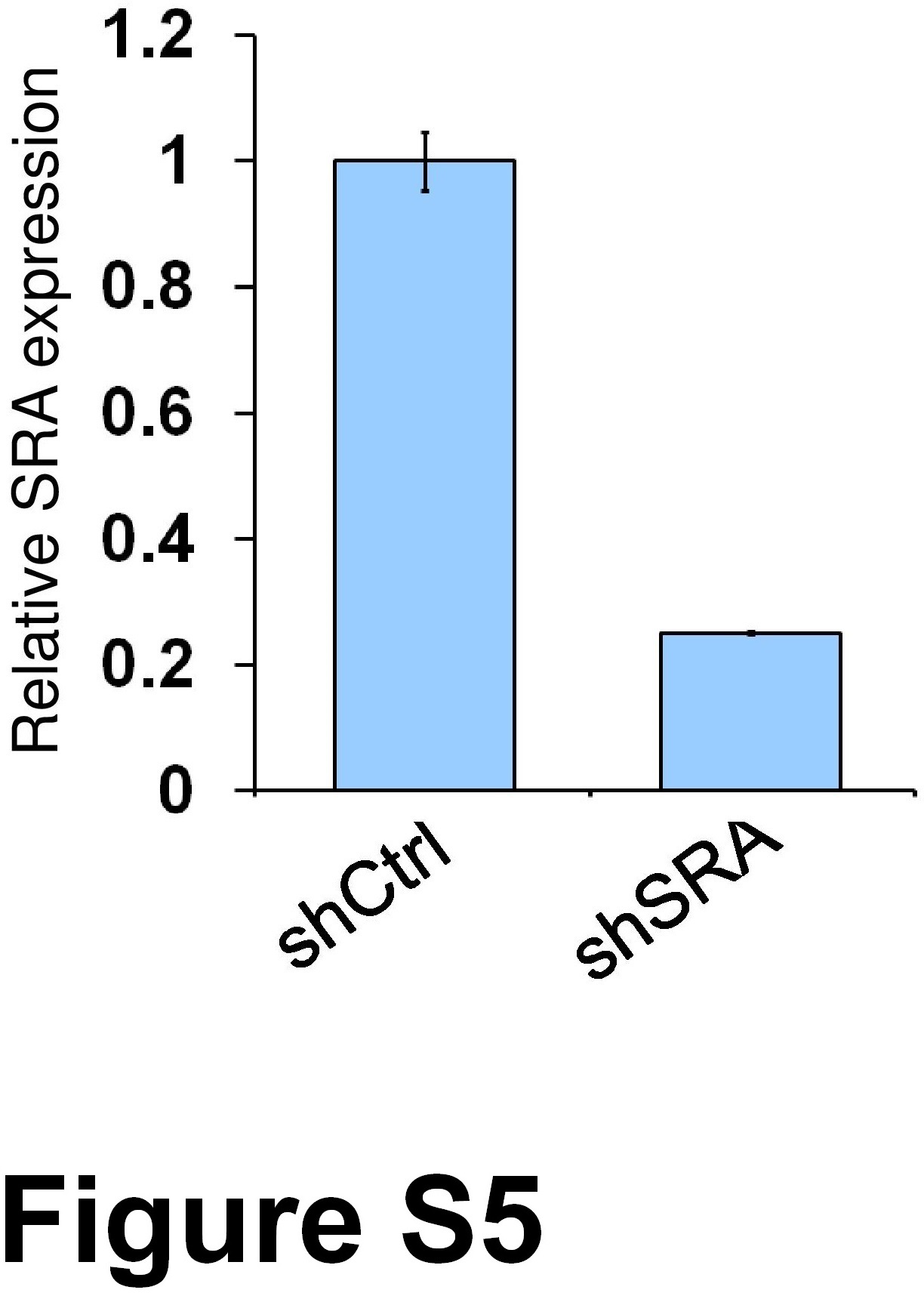

Supplement: S5 Fig — The human pluripotent stem cells NTERA2 were transfected with a plasmid encoding shRNA targeting SRA. Cells stably expressing the shRNA were established by puromycin selection. Data are shown as mean± SD; n = 3. (JPG) [file pgen.1005615.s005.jpg]

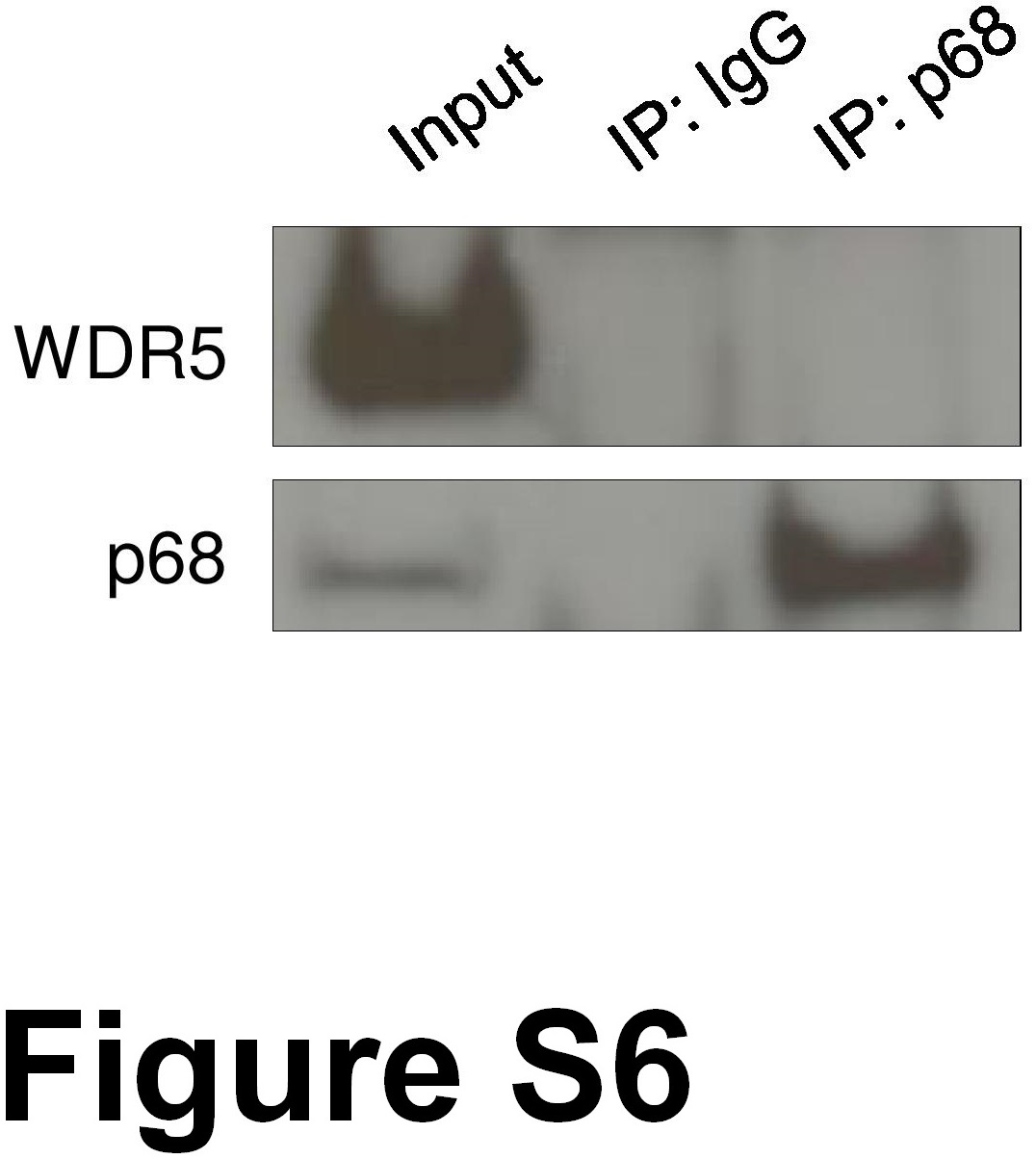

Supplement: S6 Fig — Purified recombinant TrxG complex was used for in vitro co- immunoprecipitation assay with p68. A rabbit polyclonal antibody recognizing p68 was used to pull down the RNA helicase. Western blot analysis was performed to detect p68 and TrxG interaction. The inputs were used at 10% of the samples. (JPG) [file pgen.1005615.s006.jpg]

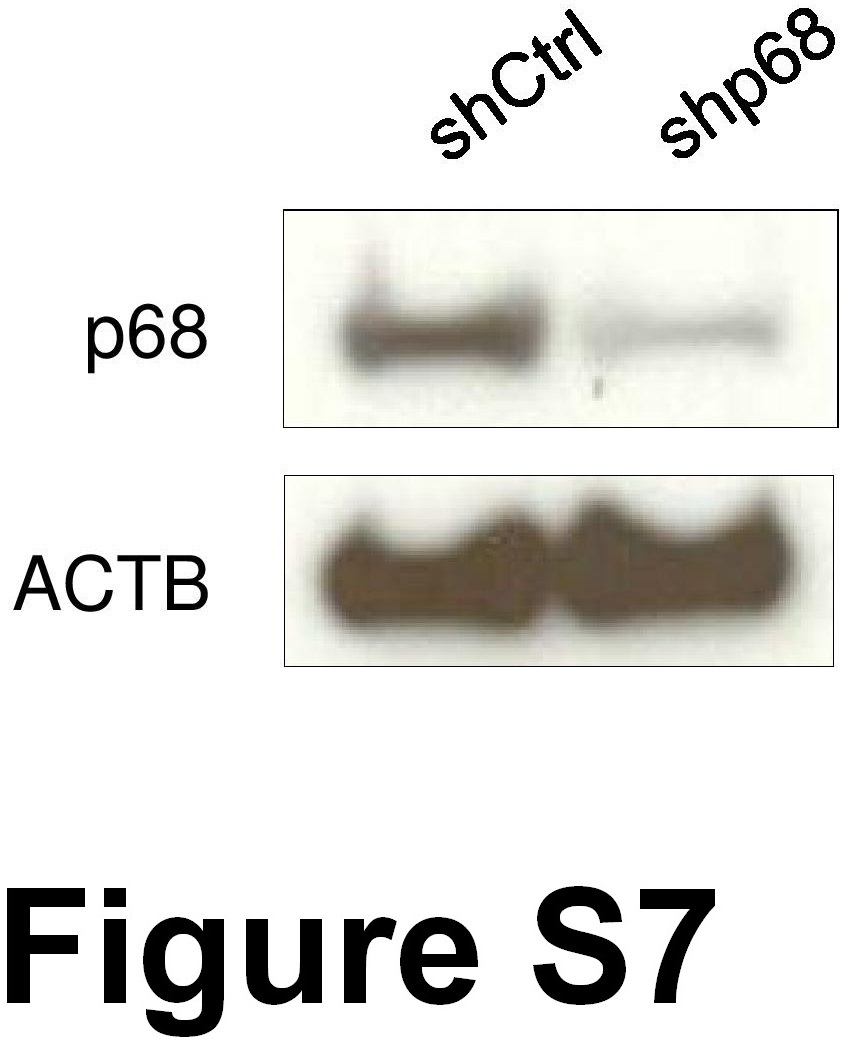

Supplement: S7 Fig — The human pluripotent stem cells NTERA2 were transfected with a plasmid encoding shRNA targeting p68. Cells stably expressing the shRNA were established by puromycin selection. (JPG) [file pgen.1005615.s007.jpg]

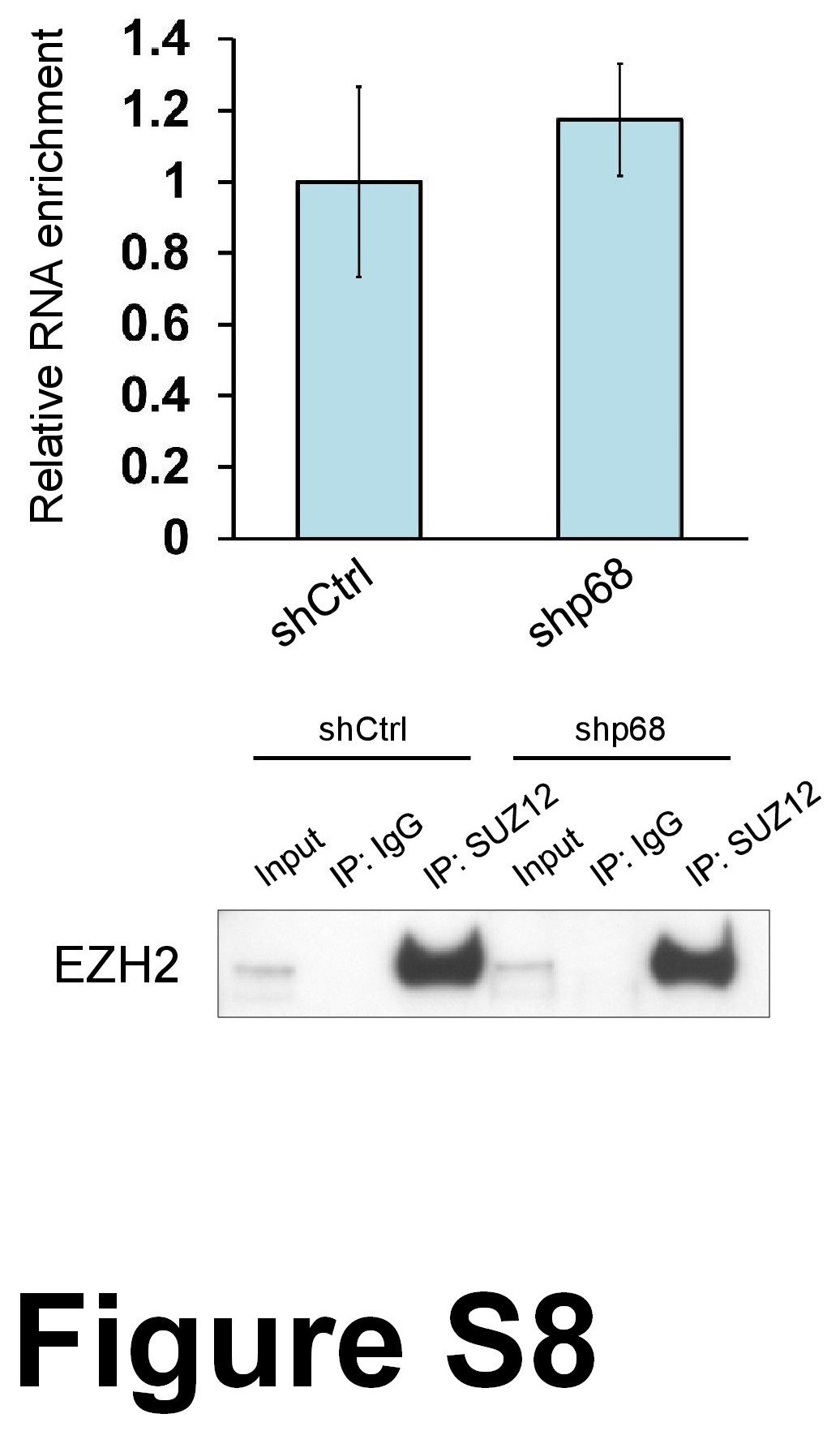

Supplement: S8 Fig — RNA immunoprecipitation was performed using scramble and p68 knockdown NTERA2 cells. Upper: qPCR of SRA purified from immunoprecipitates using anti-rabbit SUZ12 antibody. Data are shown as mean±SD; n = 3. * p < 0.05. p value calculated with two-tailed Student’s t test. Lower: Western blot of immunoprecipitates using anti-mouse EZH2 antibody. The inputs were used at 10% of the samples. (JPG) [file pgen.1005615.s008.jpg]

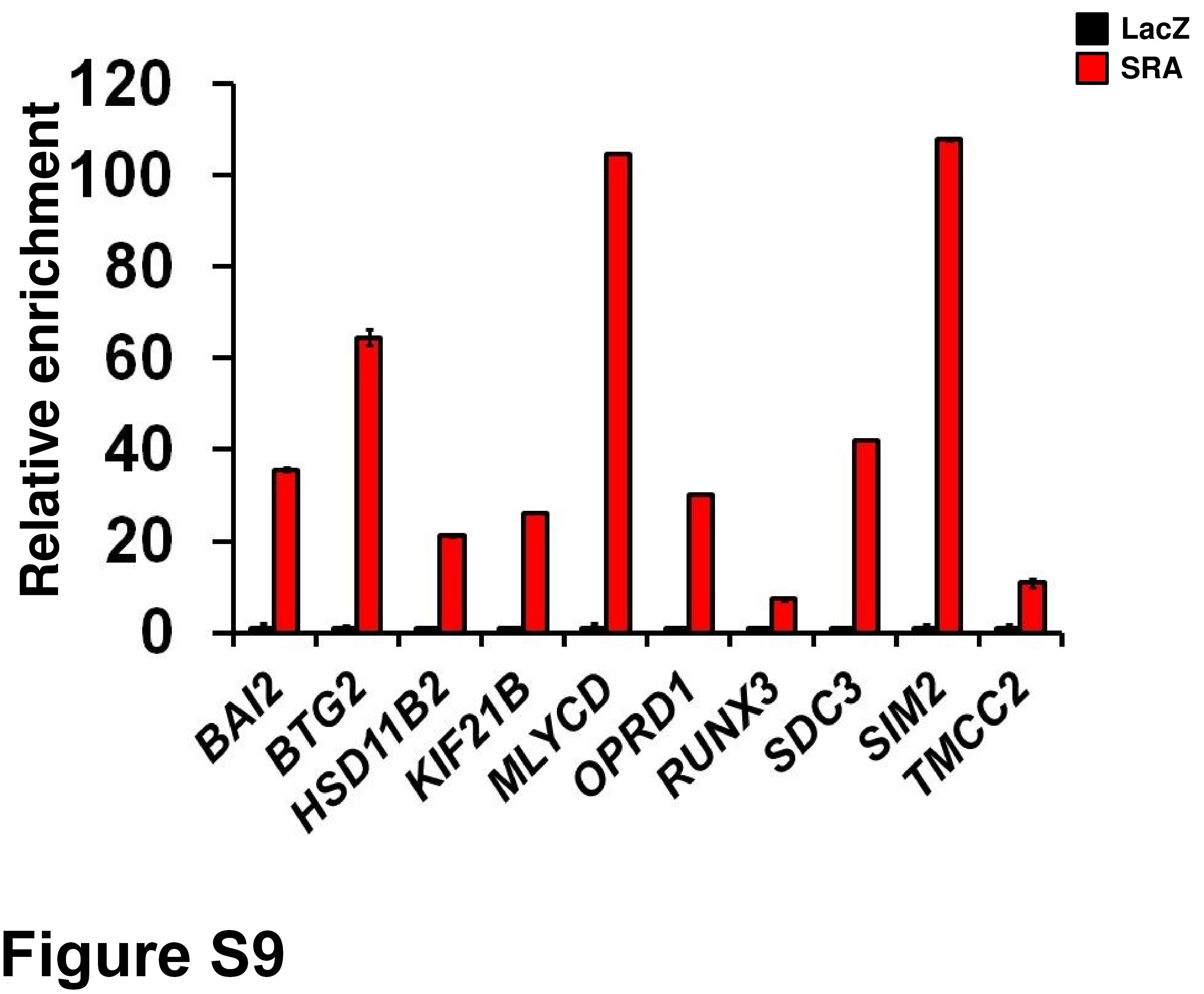

Supplement: S9 Fig — (JPG) [file pgen.1005615.s009.jpg]

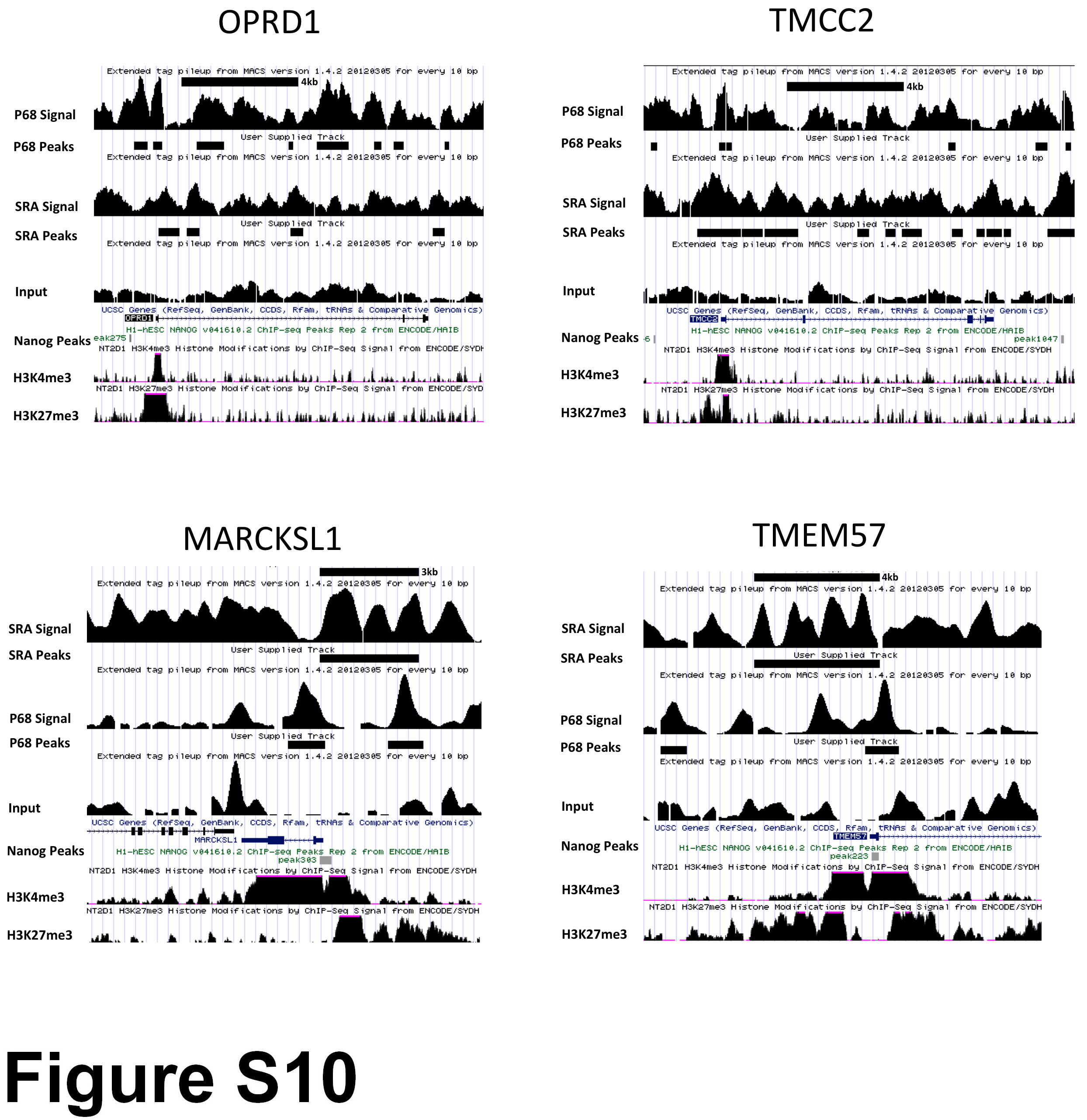

Supplement: S10 Fig — Publicly available data for H3K4me3, H3K27me3 and NANOG ChIP-seq were derived from the ENCODE project. (TIF) [file pgen.1005615.s010.tif]

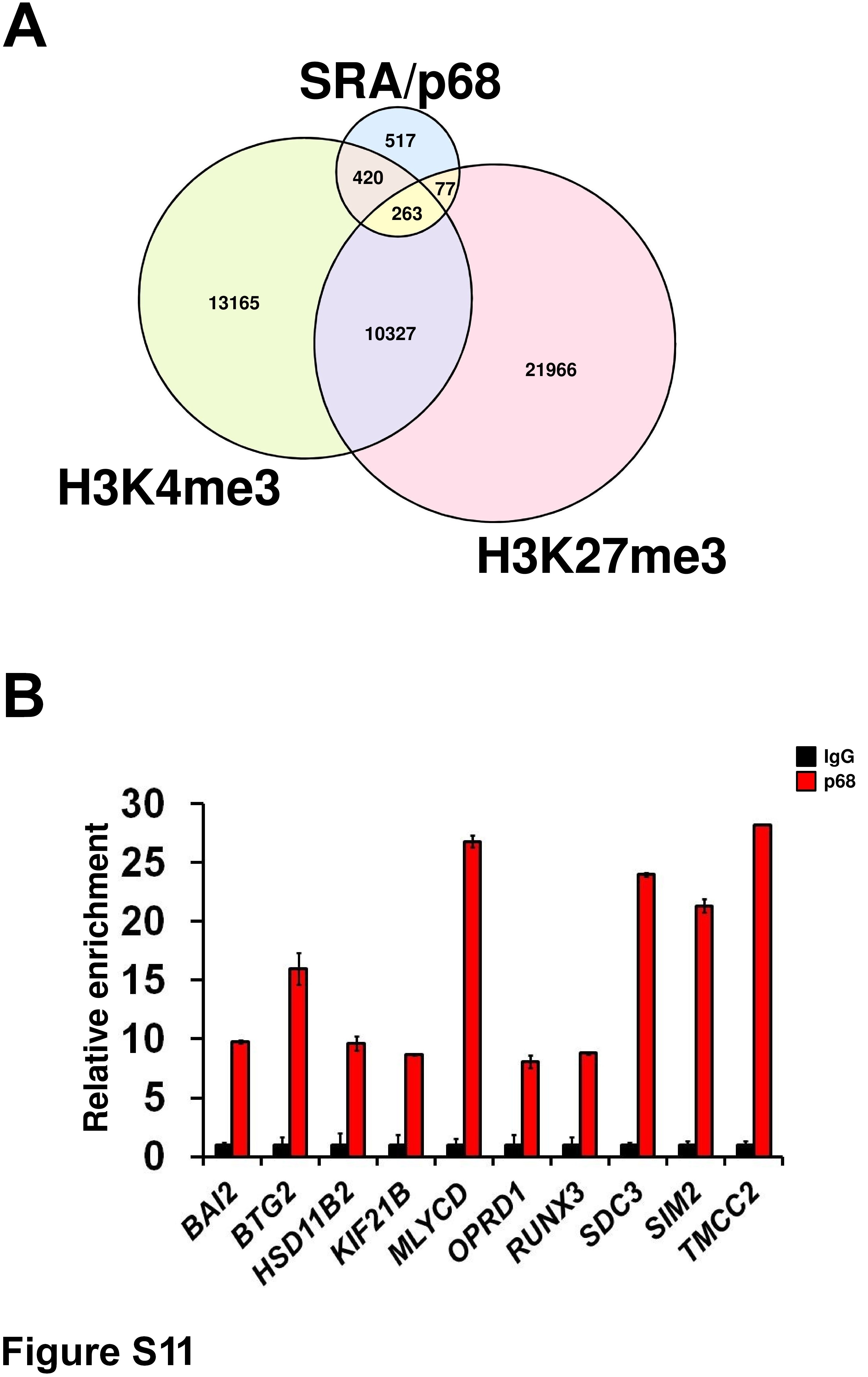

Supplement: S11 Fig — (JPG) [file pgen.1005615.s011.jpg]

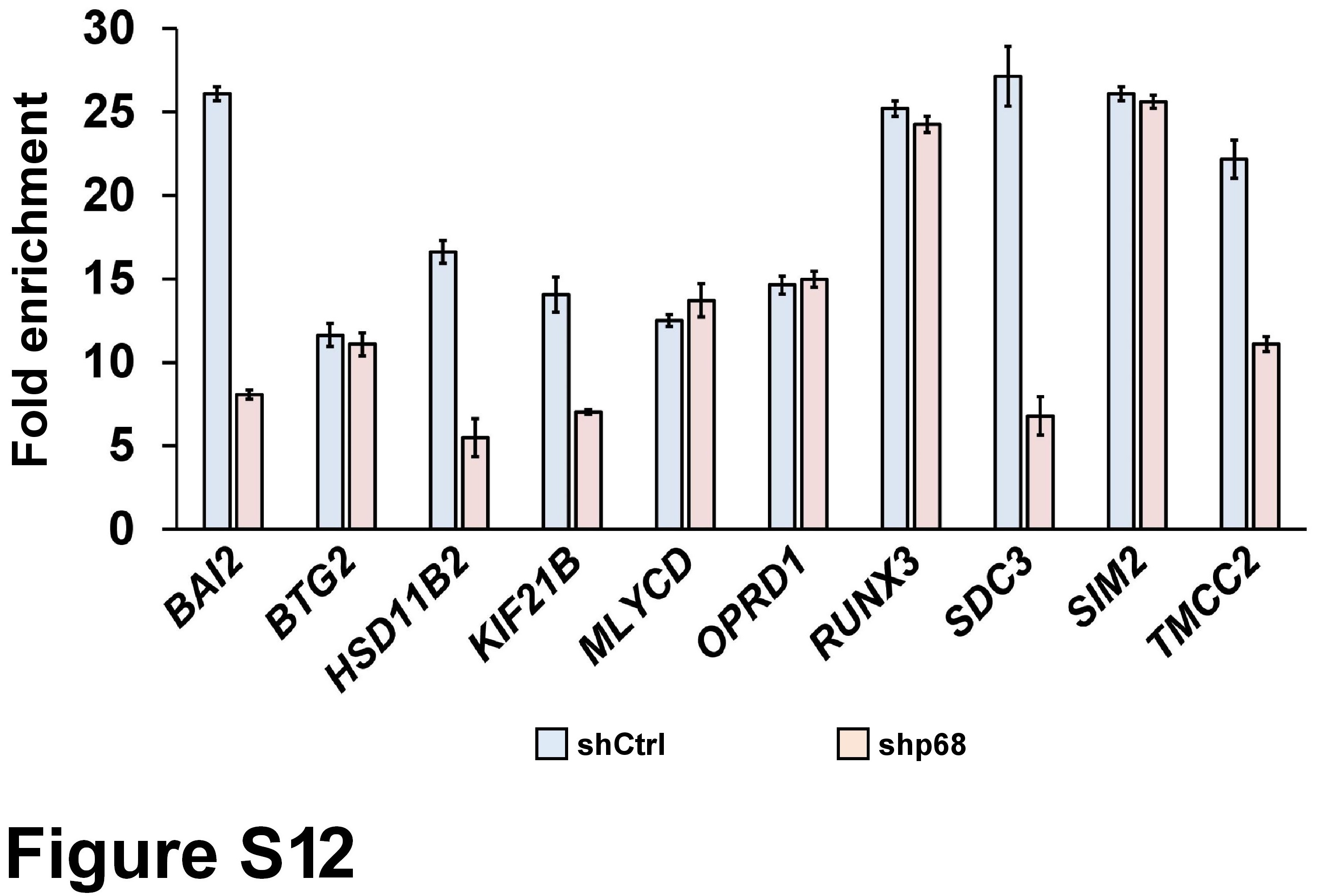

Supplement: S12 Fig — Silencing of p68 led to a decrease in H3K4me3 occupancy at a number of selected p68/bivalent target genes. (JPG) [file pgen.1005615.s012.jpg]

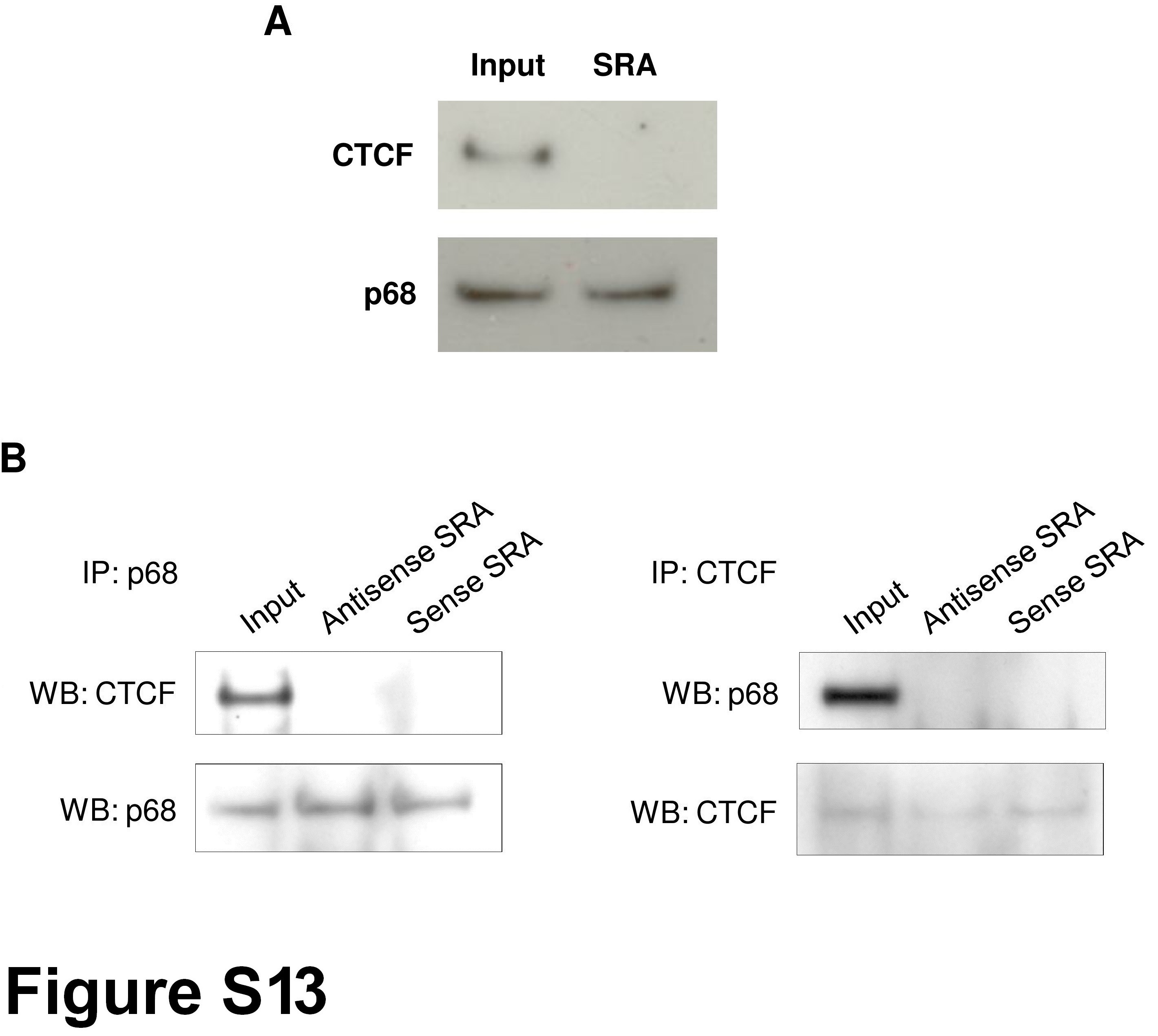

Supplement: S13 Fig — Co-immunoprecipitation (Co-IP) was performed using recombinant CTCF and p68 in the presence of antisense or sense SRA. Left: Co—IP using p68 antibody. Right: Co- IP using CTCF antibody. Note that in a previous publication (Yao et al. 20 10), although recombinant p68 and CTCF were used, the interactions were carried out in the presence of nuclear extracts. The inputs were used at 10% of the samples. (JPG) [file pgen.1005615.s013.jpg]

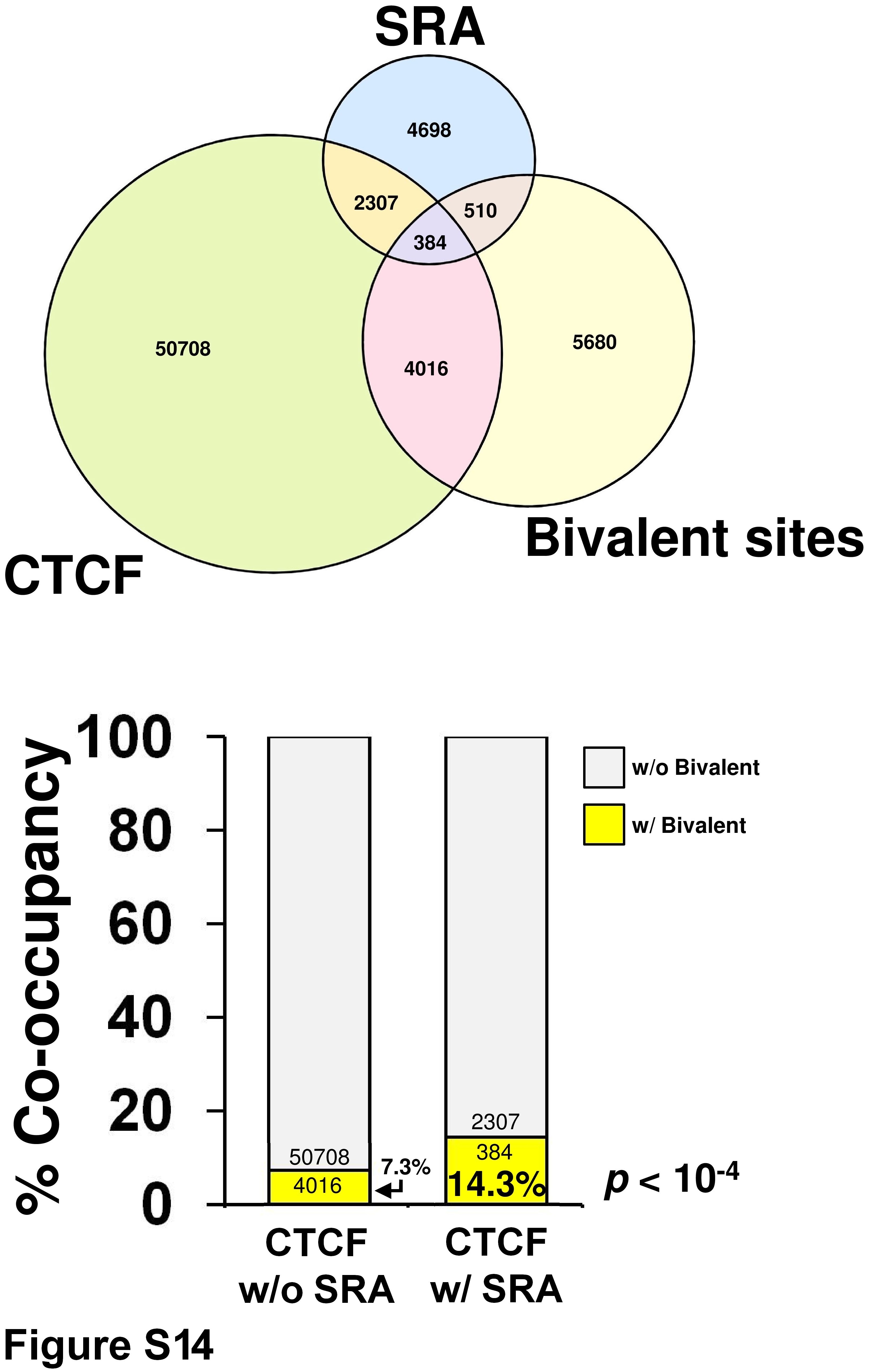

Supplement: S14 Fig — Lower; Percentage of co- occupancy of bivalent sites of CTCF binding regions without or with SRA occupancy (see text). p-value was calculated by Fisher’s exact test. (JPG) [file pgen.1005615.s014.jpg]

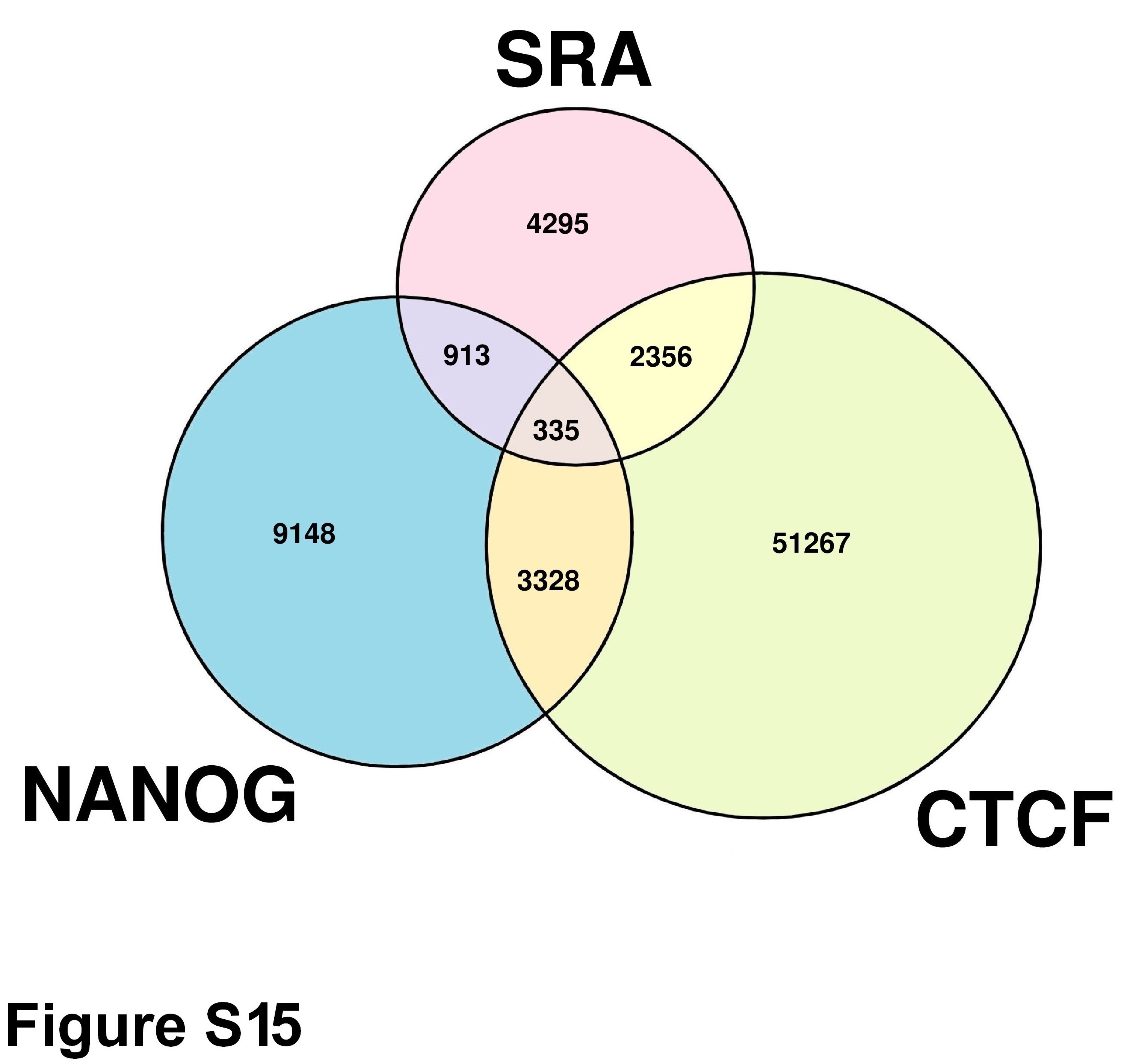

Supplement: S15 Fig — (JPG) [file pgen.1005615.s015.jpg]

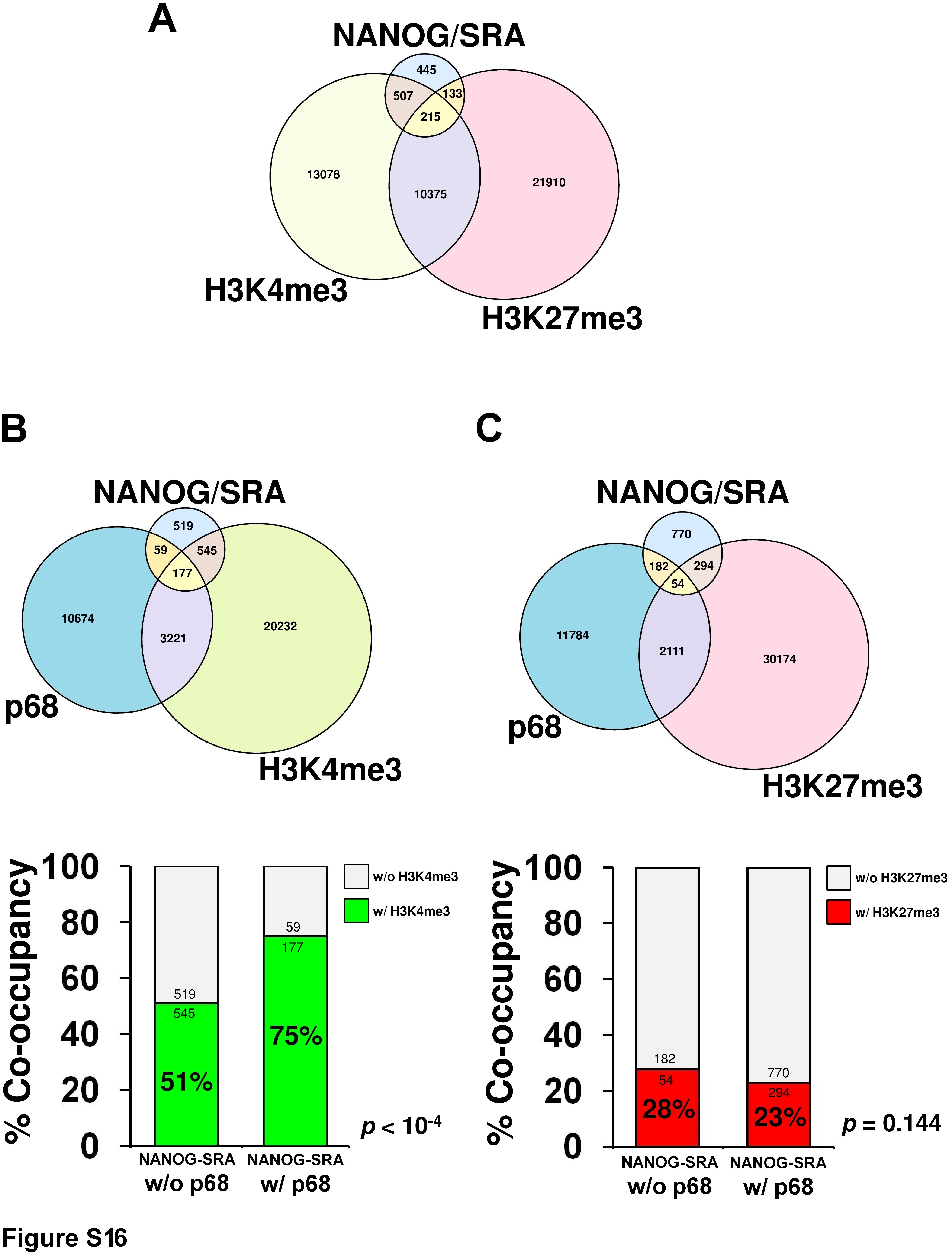

Supplement: S16 Fig — (B and C) Genome—wide p68-binding sites were compared with SRA and H3K4me3 (B) or H3K27me3 (C). Lower; Percentage of co-occupancy of H3K4me3 (B) and H3K27me3 (C) of SRA binding sites without or with p68 occupancy. p-values were calculated by Fisher’s exact test. (JPG) [file pgen.1005615.s016.jpg]

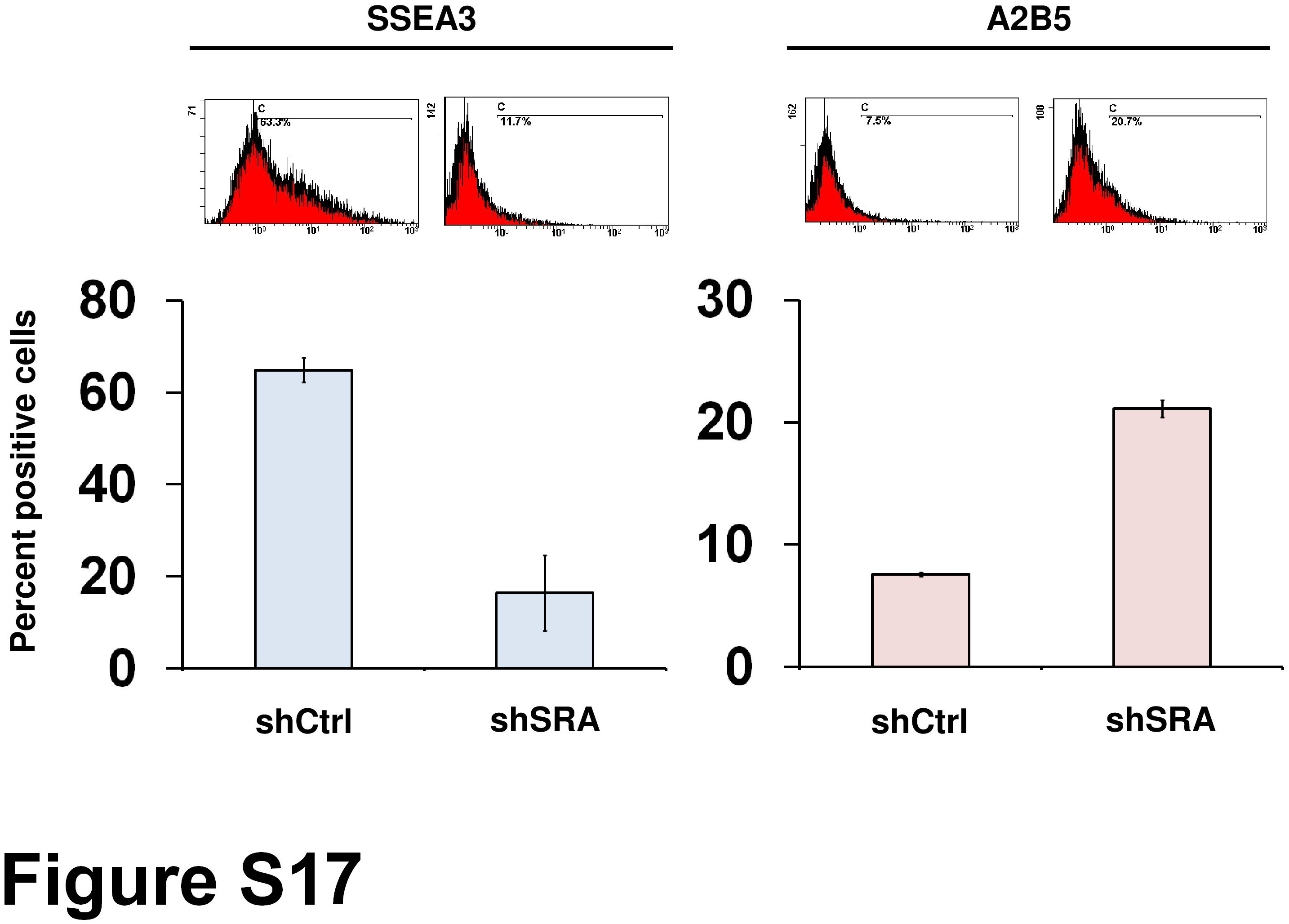

Supplement: S17 Fig — Data are shown as mean± SD; n = 3. (JPG) [file pgen.1005615.s017.jpg]

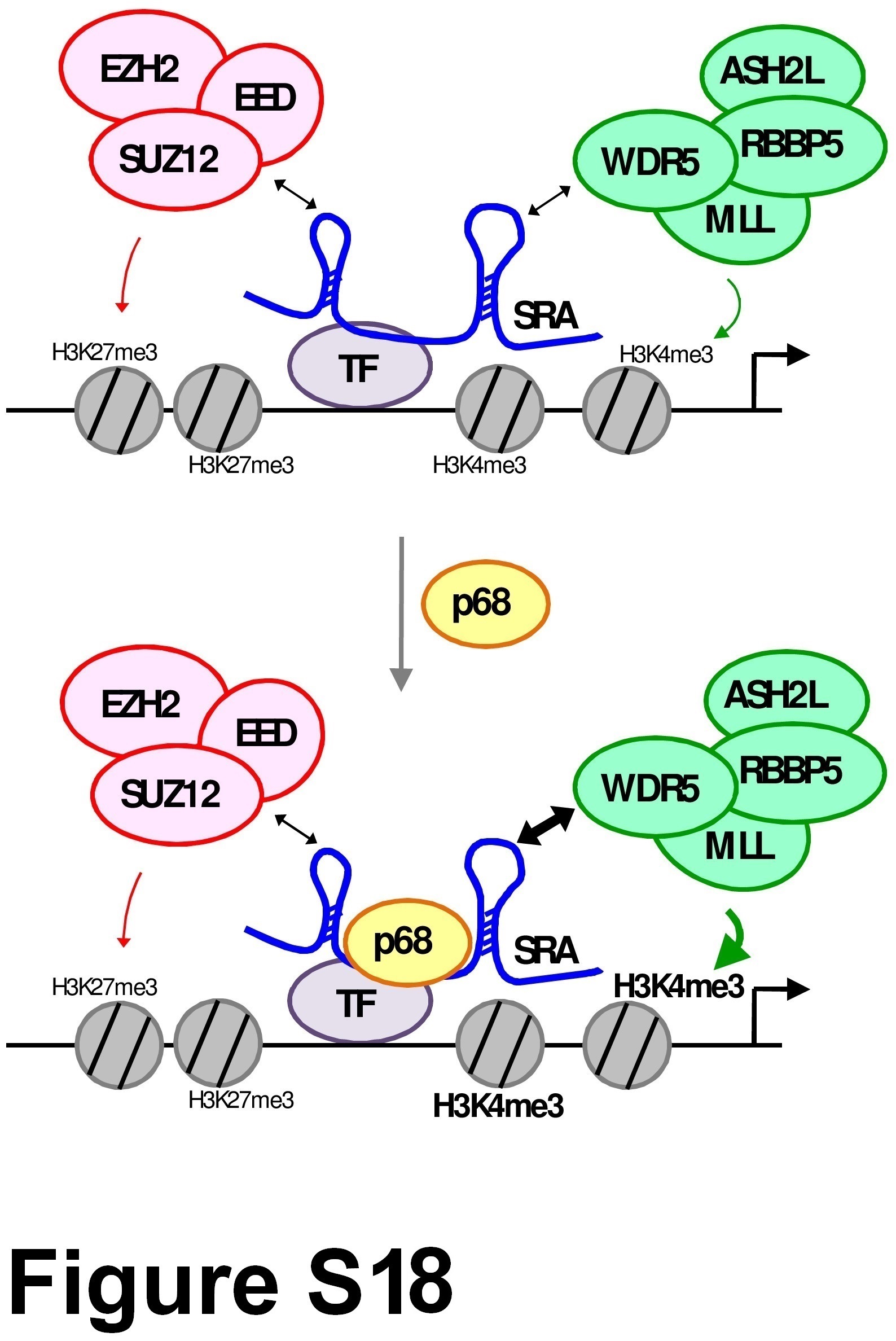

Supplement: S18 Fig — At bivalent genes harboring both H3K4me3 and H3K27me3 marks, SRA directly interacts with both TrxG and PRC2 complexes, and is recruited to target genes by transcription factors such as NANOG. The presence of p68 facilitates TrxG recruitment by SRA, which may in turn increase the level of H3K4me3. (JPG) [file pgen.1005615.s018.jpg]
