# Supplementary material for: Association of the Long Non-coding RNA Steroid Receptor RNA Activator (SRA) with TrxG and PRC2 Complexes
Source: PLoS Genet. 2015 Oct 23;11(10):e1005615. doi: 10.1371/journal.pgen.1005615 (PMC4619771; doi:10.1371/journal.pgen.1005615)
Supplement: S1 Table — (DOC) [file pgen.1005615.s019.doc]

**S1 Table**

| Gene | Species | Company | Cat. No. | Application |
| --- | --- | --- | --- | --- |
| p68 | Rabbit | Bethyl Laboratories | A300-523A | WB, IP, ChIP |
| p68 | Mouse | Santa Cruz Biotechnology | sc-166167 | WB |
| p72 | Rabbit | Bethyl Laboratories | A300-509A | WB |
| CTCF | Rabbit | Abcam | ab70303 | IP, WB |
| CTCF | Mouse | Abcam | ab37477 | WB |
| WDR5 | Rabbit | Bethyl Laboratories | A302-430A | WB, ChIP |
| RBBP5 | Rabbit | Bethyl Laboratories | A300-109A | WB, IP |
| RBBP5 | Mouse | Millipore | MABE220 | WB, IP |
| ASH2L | Rabbit | Bethyl Laboratories | A300-107A | WB |
| MLL | Rabbit | Bethyl Laboratories | A300-375A | WB |
| EED | Rabbit | Santa Cruz Biotechnology | sc-28701 | WB |
| SUZ12 | Rabbit | Santa Cruz Biotechnology | sc-67105 | WB, IP |
| EZH2 | Mouse | Thermo Scientific | MA5-15101 | WB, IP |
| NANOG | Mouse | Thermo Scientific | MA1-017 | WB, ChIP |
| OCT4 | Mouse | Santa Cruz Biotechnology | sc-5279 | WB |
| SOX2 | Rabbit | Cell Signaling | 2748S | WB |
| Control IgG | Mouse | Santa Cruz Biotechnology | sc-2025 | ChIP |
| Control IgG | Rabbit | Santa Cruz Biotechnology | sc-2027 | IP, ChIP |
| IgG+IgM FITC | Goat | Cayman Chemical | 10006617 | IF |
