# Supplementary material for: Association of the Long Non-coding RNA Steroid Receptor RNA Activator (SRA) with TrxG and PRC2 Complexes
Source: PLoS Genet. 2015 Oct 23;11(10):e1005615. doi: 10.1371/journal.pgen.1005615 (PMC4619771; doi:10.1371/journal.pgen.1005615)
Supplement: S2 Table — (DOCX) [file pgen.1005615.s020.docx]

**S2 Table.** Primer sequences for ChIP-PCR and ChIRP-PCR.

| **Primer** | **Sequence (5’→3’)** |
| --- | --- |
| BAI2_ChIPF | CATGTCACCGCGTAATCCTG |
| BAI2_ChIPR | AGCCCAATGTCCCCTTACTC |
| BTG_ChIPF | TCCCTGGACCTCCTGAAAAAC |
| BTG2_ChIPR | AAGACCTTAAGCCTCTGCTCG |
| HSD11B2_ChIPF | CTGACTCCATTGCATCGTGG |
| HSD11B2_ChIPR | TCTCCACTCATGCTCCAAGG |
| KIF21B_ChIPF | GGTAAGAACCCTGGTCTCCC |
| KIF21B_ChIPR | GGCTAGTCTTGGGCTAGGAG |
| MLYCD_ChIPF | AAGAACAGGGTTGTGGGAGT |
| MLYCD_ChIPR | CTGCCAAGACACAGACACAC |
| OPRD1_ChIPF | ATCGCATTGCCAAGTGTACG |
| OPRD1_ChIPR | CAGGATGCGGGTACTGATGA |
| RUNX3_ChIPF | AAAAGGCCACCAGAAAACCC |
| RUNX3_ChIPR | CCATATGGATCCCCAGTCCC |
| SDC3_ChIPF | TCCCTCAAGGAAAGGGGAAC |
| SDC3_ChIPR | CTTTAGGGACTGGGCTCTCC |
| SIM2_ChIPF | TGCCATACCCGTGAACATCT |
| SIM2_ChIPR | CATTAGCCAGCTTCCGACAC |
| TMCC2_ChIPF | ATGGGTGGAGGAAATCCTGG |
| TMCC2_ChIPR | GTTTTTGGGGTTGGTTGCAC |
