# Supplementary material for: Association of the Long Non-coding RNA Steroid Receptor RNA Activator (SRA) with TrxG and PRC2 Complexes
Source: PLoS Genet. 2015 Oct 23;11(10):e1005615. doi: 10.1371/journal.pgen.1005615 (PMC4619771; doi:10.1371/journal.pgen.1005615)
Supplement: S3 Table — (DOCX) [file pgen.1005615.s021.docx]

**S3 Table.** Sequences of ChIRP probes for SRA

| **Probe** | **Sequence (5’→3’)** |
| --- | --- |
| SRA_1 | GGCTGATGTCATCACATACC/3BioTEG/ |
| SRA_2 | GGTATTGACAACTTTCCTCC/3BioTEG/ |
| SRA_3 | CGGTGGCTTGAAAGCTCTTG/3BioTEG/ |
| SRA_4 | GTCACATGGTCAACCATGAG/3BioTEG/ |
| SRA_5 | AGACTCCTCTTTTCTGCAAT/3BioTEG/ |
| SRA_6 | CTGTGGCTGCAGATTTCTCT/3BioTEG/ |
| SRA_7 | GAACCGAGGATTATGAAGCC/3BioTEG/ |
| SRA_8 | AGAAGGTCTCCAAGGCATAG/3BioTEG/ |
| SRA_9 | CCAGTGGGACAGTCTTGGTG/3BioTEG/ |
| SRA_10 | TGAGTAACACATGAACTCCC/3BioTEG/ |
| SRA_11 | ACTGTTAGCTTATACTGGGG/3BioTEG/ |
| SRA_12 | TGTTGCCTGCGGAGGCGAGG/3BioTEG/ |
| SRA_13 | CAGCCCGTATGAGAACTGCG/3BioTEG/ |
| SRA_14 | CTCTTGGTAAGCAGCGAGCG/3BioTEG/ |
| SRA_15 | TCTCTGATGCGGGGACTCTG/3BioTEG/ |
| SRA_16 | TACTTGAAGGAGGTGGAGGC/3BioTEG/ |
| SRA_17 | CGCCAGAGGCAGGACCACTC/3BioTEG/ |
| SRA_18 | CTCCATCACAGCCTCAGACT/3BioTEG/ |
| SRA_19 | GCCACGGCAGTCTTCCAATG/3BioTEG/ |
| SRA_20 | CTAGCTTGGCACCGGAAGGG/3BioTEG/ |
| SRA_21 | TGCAGATACACAGGGAGCAG/3BioTEG/ |
| SRA_22 | CAGCAGATCCCTTCCTGATG/3BioTEG/ |
| SRA_23 | AGTCTACTTTGGGAAAAAGT/3BioTEG/ |
| SRA_24 | ACCATCCAAACACAGCTGCT/3BioTEG/ |
| SRA_25 | AAGTCTCCCCGTTCTATTTG/3BioTEG/ |
| SRA_26 | GTGATACAGTGCAAATAGGA/3BioTEG/ |
| SRA_27 | CCACTCAATGTGGTTCTAAT/3BioTEG/ |
| SRA_28 | CTCAGTAATCTGGTCTTTTT/3BioTEG/ |
| SRA_29 | TGAGGCAGCAGTTTGTTTGG/3BioTEG/ |
